# Defending Airports from UAS: A Survey on Cyber-Attacks and Counter-Drone Sensing Technologies

**DOI:** 10.3390/s20123537

**Published:** 2020-06-22

**Authors:** Georgia Lykou, Dimitrios Moustakas, Dimitris Gritzalis

**Affiliations:** Department of Informatics, Athens University of Economics & Business (AUEB), GR-10434 Athens, Greece; lykoug@aueb.gr (G.L.); dmoustakas@aueb.gr (D.M.)

**Keywords:** unmanned aerial vehicles (UAV), counter unmanned aerial systems (C-UAS), sensors, sensing technologies, drones, cyber-physical systems, airport resilience, critical infrastructure protection

## Abstract

As the fastest growing segment of aviation, unmanned aerial systems (UAS) continue to increase in number, technical complexity and capabilities. Numerous civilian and commercial uses are drastically transforming civil protection, asset delivery, commercial and entertaining activities. However, UAS pose significant challenges in terms of safety, security and privacy within society. An increasing phenomenon, nowadays, is drone-related incidents near airport facilities, which are expected to proliferate in frequency, complexity and severity, as drones become larger and more powerful. Critical infrastructures need to be protected from such aerial attacks, through effective counteracting technologies, risk management and resilience plans. In this paper, we present a survey of drone incidents near airports and a literature review of sensor technologies, able to prevent, detect, identify and mitigate rogue drones. We exhibit the benefits and limitations of available counter-drone technologies (C-UAS); however, defending airports against misused drone activity is a hard problem. Therefore, we analyze three realistic attack scenarios from malicious drones and propose an effective C-UAS protection plan for each case. We discuss applicability limitations of C-UAS in the aviation context and propose a resilience action plan for airport stakeholders for defending against airborne threats from misused drones.

## 1. Introduction

Unmanned aircraft systems (UAS), unmanned aerial vehicles (UAV), and remotely piloted aircraft systems (RPAS) are all different ways of referring to what are most commonly known as drones [1]. They provide a game-changing technology, transforming commercial industries, media and entertainment, while future opportunities in the field are limitless. A decade ago, drones were considered a technology restricted only to official authorities, such as the military, police, etc. However, many sectors have begun to use UAVs for delivering goods and services. The US Federal Aviation Administration (FAA) predicts that more than two million drones will be operated in the USA by 2020 [2]. On the other hand, UAS pose a significant challenge in terms of safety, security and privacy for our society and many drone-related incidents are frequently reported affecting critical infrastructures (CIs), especially around airport facilities. According to the United Nations Security Council, the increased accessibility to drones, combined with their technological evolution, has led to renewed attempts by malicious actors, including organized crime and terrorist groups, who exploit UAS for nefarious purposes [3]. There have been several examples of terrorists using weaponized UAS to conduct attacks, or support surveillance, reconnaissance and other illegal activities. These incidents have created the need to detect and disable rogue drones; therefore, a new area of research and development has emerged in counter-drone technologies (C-UAS) [4].

UAVs are multi-rotor or fixed-wing aircrafts, autonomously piloted or operated by a remote controller. They come in many shapes and sizes, ranging from insect-like types to large ones that weigh several tons. Different organizations (NATO, DoD, NASA and regulatory authorities) have defined main UAS categories. Most of these classifications are based on weight, operating altitude or speed. While classification group nomenclature differs among these organizations, some specific weight limits are commonly used, as presented in Table 1.

Table 1 shows UAV categorization based on weight, altitude, range, endurance and payload capabilities, along with some examples of available commercial UAV models. In this work, we analyze threats from the lightweight class of micro, mini and small UAVs (sUAS, NATO Class I) weighing less than 150 kg. Such drones can navigate quite far from the center of command, up to a range of 50 km, with an average speed of 15 m/s. They are able to carry heavy payloads up to 50 kg and provide video piloting and a communication link based on radio signals. There are two types of technologies for command and control (C2) communication: Wi-Fi and analog. Moreover, a live video stream can be sent from the drone’s video camera to the pilot (operator) via a GCS (ground control station), which can be a dedicated controller, smartphone, VR glasses, etc. [5]. For this category of commercial-off-the-shelf sUAS, new innovative control interfaces have been recently developed. The emerging field of human–drone interaction (HDI) was surveyed by Tezza and Andujar [6], who discussed how HDI goes beyond control modalities, enhancing human interaction.

The recreational and commercial uses of drones have expanded in evolving smart cities, where UAVs perform multiple activities. Alsamhi et al. [7] have reviewed the collaboration of drones and the Internet of Things (IoT) for improving intelligence and quality of life in smart cities. Moreover, in rural areas and critical infrastructures, new uses for UAVs fulfill operational, safety and environmental monitoring tasks, which include taking physical, chemical, electromagnetic and radiochemical measurements. They extend human safety capabilities, by monitoring environments where humans cannot reach [8,9]. In the security sector, UAVs can expand the deployment of traditional security detection (e.g., sensors and cameras) with perimeter monitoring systems in CIs, including airport facilities. Furthermore, within the airport perimeter, UAS can provide a faster response to security alarms, track threats, and inspect or patrol facilities, as presented in [10].

**Figure 1:**
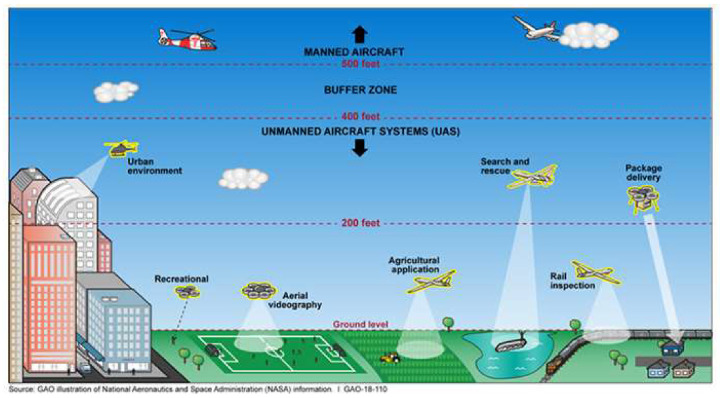
Potential civilian and commercial uses for small UAS [11].

Figure 1 graphically represents potential civilian and commercial uses for UAVs and interactions with aviation activities, as published in the GAO report [11]. In the drawing, we can distinguish drones performing package delivery, aerial videography and other recreational activities in urban areas. They are also used in agricultural applications and critical infrastructure inspection in rural areas and support search and rescue activities, while flying in lower airspace up to 400 feet from ground level.

Although advances in UAV technology have found numerous applications and brought multiple benefits to society in general, the potential threat of technology misuse should not be discounted. Nassi et al. [5] describe societal threats to security and privacy created by drones, while Altawy and Youssef [12] have identified both physical and cyber threats from such systems. In our research, we have distinguished asymmetric threats, which can exploit sUAS capabilities to attack CIs, including airports, in an obscure or unusual fashion, providing unfair advantage to the perpetrator. These have been aggregated into the following three categories:

- Spying and tracking points of interest, conducting unauthorized mapping and surveillance.
- Carrying CRBNE payloads (chemical, radiological, biological, nuclear and explosive materials) towards fixed installations or moving targets.
- Intercepting wireless networks, breaching computer systems and conducting cyberattacks by hovering or landing on buildings.

In this paper, we present a survey on drone incidents and countermeasures, focusing our research on airport facilities and surrounding critical infrastructure. We continue with a literature review on C-UAS sensors and technologies, which includes both academic publications and industry developments in countering drone systems. Benefits and limitations of each detecting and counteracting technology are also presented, along with applicability challenges of C-UAS systems in the complicated aviation environment. In addition, we have developed various attack scenarios against airport CIs with the use of UAVs, based on the three categories of asymmetric threats listed above. A detailed description for each scenario is presented, along with affected assets and impact analysis. Graphical attack representations depict malicious attack phases, on a step-by-step basis, while related impacts on security parameters are also examined. Finally, we propose prevention measures, along with detection and mitigation technologies, which could be deployed in each scenario, in order to counteract and protect airport CIs from UAS malicious attacks.

The principal aim of our research is to develop an overview of the available risks from misused UAVs and make recommendations for the design of effective C-UAS in airport facilities. To the best of our knowledge, no study has presented analytical attack scenarios, which can be launched inside and outside airport perimeters (in eight potential attack-launching spots). For each attack scenario, a proposed C-UAS protection plan is designed, aiming to increase airport resilience and business continuity.

This article’s contribution is to: (i) alert the airport community and aviation researchers about safety and security risks from nefarious use of drones, (ii) analyze benefits and limitations of available C-UAS technologies and (iii) propose a resilience action plan that supports airport operators and aviation stakeholders to increase the robustness of critical assets and infrastructures against airborne malicious threats.

The remainder of this paper is structured as follows: In Section 2, we present our survey on worldwide drone incidents, threatening airports and critical infrastructures. In Section 3, we review methods and sensor technologies able to detect, identify and mitigate rogue drones. In Section 4, we analyze three different categories of attack scenarios against aviation assets and airport CIs, while in Section 5, a C-UAS protection plan is proposed for each scenario. Finally, in Section 6, we discuss applicability limitations of C-UAS in the aviation context and propose a resilience action plan for defending airports from misused drones. Findings and conclusions of our research are presented in Section 7.

## 2. Worldwide Incidents with UAS

Since 2016, the number of security incidents involving UAVs near airports and other CI facilities has dramatically increased worldwide. It is obvious that drones can pose a potentially severe threat to aviation activities [10,11,12]. The major problem with drones operating near airports and air-controlled space is the collision hazard between manned aircrafts and drones, which raises the safety risk of human and material losses. Tests conducted by the UK government found that a 400-g drone could smash a helicopter’s windscreen, while a 2-kg drone could cause critical damage to a passenger jet’s windscreen [13]. While cheap UAV versions have barely enough power to fly for half an hour, sophisticated models can stay airborne for hours at a time. As a result, whenever an unauthorized drone is detected around an airport and its facilities, in the vicinity of runways, or even close to the security perimeter, the entire airport may be closed for safety reasons. This is translated into unnecessary costs, time delays and potentially a negative reputation for the airport, air traffic control (ATC) and the Civil Aviation Authority. In this section, we present our survey of UAV incidents affecting aviation activities, which includes UAS sightings and verified UAV incidents near airport facilities with quantified impacts.

Although UAS incident reporting is not mandatory yet, we have examined and exhibited drone incidents witnessed over the last 4 years by using open, publicly available sources and databases that report UAVs incidents, like NASA, FAA, Dedrone, ASN and others [14,15,16,17,18]. We have distinguished 10 serious incidents in heavy traffic airports worldwide, with serious impacts on safety, security, reputation and quantified economic loss, and we present this collection of events below:

1. UK: A serious incident happened between 19–21 December 2018 in London, when Gatwick Airport stopped its operations due to a drone attack. Police investigators said that it was a planned attack, involving someone with inside knowledge of the airport’s operational procedures. It is estimated that 140,000 passengers were affected, with around 1000 flights either diverted or cancelled [19]. The attack cost the airport approx. £1.4 m, but airlines were hit even harder, with EasyJet is said to have lost £15 m through the 3-day attack. A similar disruption took place one month later at Heathrow Airport in January 2019, although with limited duration [20].
2. Ireland: Flight operations at Dublin airport were suspended for half an hour in February 2019 due to the confirmed sighting of a drone over the airfield, despite a drone prohibition within 5 km (3 m) of Irish airports [21].
3. Germany: Frankfurt airport was shut down for an hour on 9 May 2019 as operators halted flights over a drone sighting. Overall, 143 take-offs and landings were cancelled, while 48 aircrafts were diverted to other airports among a total of 1500 scheduled flights [22].
4. Singapore: Two incidents occurred where unauthorized drone flying affected flights at Changi Airport twice in one week during June 2019. Overall, 52 flights were delayed and 8 were diverted due to these drone sightings [23].
5. UAE: Dubai International Airport (DXB) was closed three times (an accumulated 115-min closing) in 2016 due to illegal drone activities near the airport. The Emirates Authority for Standardization and Metrology estimated the financial losses to be USD 95,368 per minute due to shutdowns caused by drones. The total loss of DXB in 2016 was approx. USD 11M due to drones [24].
6. Japan: A drone spotted flying near Osaka’s Kansai International Airport in October 2019 led to the temporary closure of the major hub, despite the fact that flying drones near Kansai Airport and bringing drones inside the airport is prohibited [25].
7. Canada: A Beech King Air A100 of Skyjet Aviation collided with a UAV in October 2017 while approaching Jean Lesage Airport near Quebec City. The aircraft landed safely despite being hit on the wing. Neither the UAV nor the operator have been found. The UAV had been flying at 1500 ft, i.e., five times the maximum altitude that UAVs are permitted to fly at in Canada [26].
8. New Jersey, USA: Newark Airport was closed due to a drone spotted in the vicinity for 90 min in January 2019. Estimating a cost of USD 1M per minute for the airport closure, the incident caused USD 90M of economic loss. Airplanes were diverted to other airports using extra fuel consumption and adding to the economic loss for the airlines [27].
9. New York, USA: A civilian UAV collided with a Black Hawk helicopter over the eastern shore of Staten Island in September 2017. The helicopter was able to continue flying and landed at Linden Airport. Nobody was hurt, but part of the UAV was found at the bottom of the main rotor system [28].
10. South Carolina, USA: A helicopter crash was triggered by a civilian drone in February 2018. This was the first drone-linked aircraft crash. The helicopter’s tail struck a tree while trying to evade a small drone, triggering a crash landing. The student and instructor pilots were uninjured, according to a Charleston Police Department Report [29].

**Figure 2:**
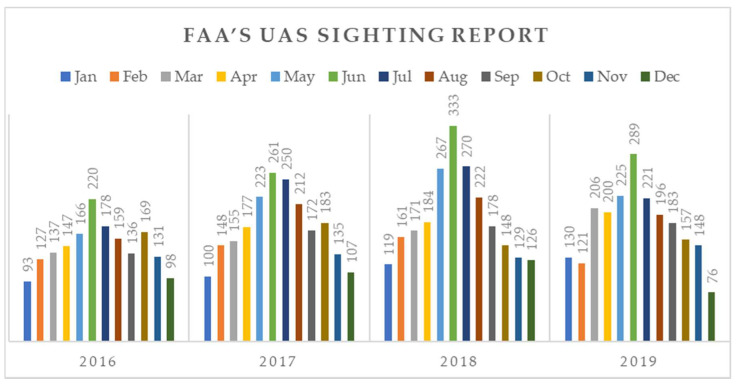
FAA’s UAS sighting report database.

In addition to the above incidents with quantified impacts, encounters and near misses between manned and unmanned aircrafts are becoming increasingly common events, despite the existing restrictions around air-controlled spaces and geofencing measures. Since 2016, the US Federal Aviation Administration (FAA) has collected 8344 reports from airmen about UAS sightings of potentially unsafe use [15]. In Figure 2, these sightings of non-compliant UAS operations are graphically analyzed, where we can distinguish the occurrences’ seasonality in summer months and the gradual annual increase in sightings.

Despite the FAA’s efforts and initiatives to regulate and contain the risks of unsafe or non-compliant sUAS operations in the aviation sector, the problem seems to be accelerating, with more than 2000 near miss sightings per year being reported by airplane pilots, air traffic controllers and other aviation stakeholders [30]. However, these events cannot be outright verified, so to be reported as incidents, thus the FAA’s UAS Sighting Report Database provides a barometer of unsafe UAS operations [15].

The increasing number of occurrences near airports has led to serious safety concerns being raised for drone violations of aviation safety rules. All these occurrences, combined with the rapid development of UAV technology and the uncontrolled spread of drone usage, have motivated our survey for counter-drone sensing technologies and methodologies proposed by the academic sector and applied by industry.

## 3. Literature Review on Counter-Drone (C-UAS) Technologies

The need to protect critical infrastructure from misused drones has brought advances in C-UAS academic research and commercial applications. Countering a drone is a complex, multi-step process, involving interaction between several distinct sensors and methodologies, along with interaction with human operators. In this section, we provide a literature review of major C-UAS sensor technologies that can be used in airports, classified into three main categories: (i) preventing actions, (ii) detection sensors and technologies and (iii) mitigation countermeasures against rogue drones.

Since 2014, increasing interest in C-UAS academic research has led to more than 950 scientific publications. In Table 2, the number of new publications which included the term “C-UAS” in their title is presented, excluding patents and citations, based on a Google scholar search [31]. Moreover, from January and up until March 2020, another 47 related publications were published. This proliferating trend in the number of scientific publications confirms the growing stimulus of the research community in this area, which is related to drone detection sensors and mitigation technologies.

### 3.1. Preventing Actions

The use of UAS is generally regulated by national civil aviation authorities and national institutions. In many countries, legislation proposes a set of rules to control the effects of small UAVs on peoples’ safety, security and privacy [1]. Such regulation frameworks, complemented by geofencing technologies, can act as preventive measures to forestall drone operators from entering restricted airspace by mistake or by ignorance. 

Geofencing is the creation of virtual fences around areas or points of interest to keep drones away from no-fly zones [32]. Substantial work has already been published to define and realize geofencing systems for small UAS [32,33,34,35,36]. Popular autopilot systems currently offer simple containment volume geofences over critical areas, including airports [33]. A geofence could be dynamically generated, as in a radius around a location point with predefined set of boundaries [34,35,36]. It is an effective preventing measure when built into a UAV’s navigation software [37]. As a result, drones using Global Positioning System (GPS) or Global Navigation Satellites Systems (GNSS), combined with autopilot software, can interact with a geofence and avoid restricted areas [38].

**Figure 3:**
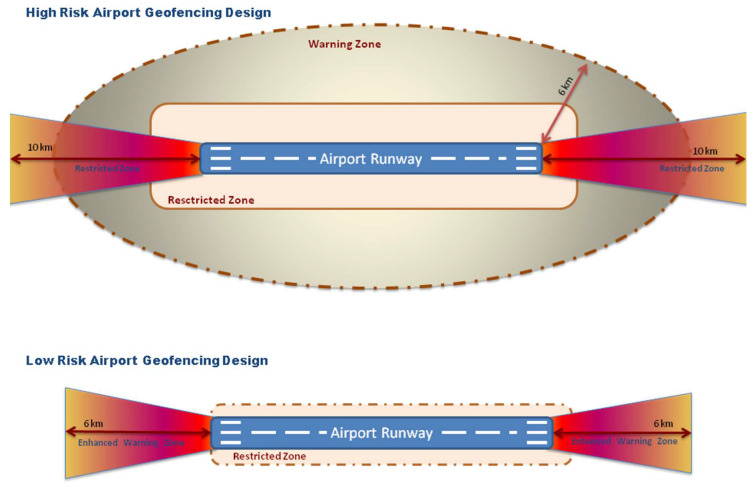
Detailed three-dimensional geofencing solutions around airports [39].

Furthermore, geofencing software can be regularly updated by UAV manufacturers to include new and temporary restricted zones. Some manufacturers have also expanded the airport restricted zones to enhance safety zones, which prevent UAVs from entering into a three-dimensional bow-tie geofence shape [39]. This protects approach and departure pathways and prevents misused drones from interfering with airplanes while departing from or landing at airports. Risk-based airspace principles can categorize airports’ virtual fences into high- and low-risk designs, as presented in Figure 3. In lower-risk areas, drone operations may be permitted, for example, when authorized UAVs are allowed to conduct drone inspection activities in locations parallel to runways.

Geofencing can play a major role in ensuring that careless and clueless UAS operators are prevented or alerted when interfering with airport airspace. However, it cannot stop malicious users from manually disabling UAV geofencing functionality in order to intrude into restricted areas. Therefore, detecting and interdicting C-UAS measures for nefarious drones in no-fly zones are also needed.

### 3.2. Detection Sensors and Technologies

In this subsection, we present a literature review on detection sensors and technologies using various types of sensors, like (i) radar detection sensors; (ii) radio-frequency detection sensors; (iii) acoustic sensors; and (iv) visual sensors. Afterwards, we make a comparison of the benefits and limitations of each technology sensor, while commercially available detection systems are also exhibited and analyzed in the last part of this section.

**Radar Detection**

A surveillance radar is designed with single or multiple antennas to detect and track multiple objects simultaneously. It sends out a signal in order to receive aircrafts’ reflection, measuring spatial coordinates and, optionally, velocity, acceleration and direction. According to Skolnik [40], no other sensor can measure range to the accuracy possible with radar at such long ranges and under adverse weather conditions. In recent years, there has been an active area of research in the field of C-UAS radar applications. Monostatic radars work with a collocated transmitter and receiver. Several studies analyzed a monostatic radar working either at 35 GHz [41] or at 9.4 GHz [42] to detect and track nearby drones. The most employed radar signal characteristic for automatic target classification is the micro-Doppler (m-D) signature [43,44]. The intrinsic rotation movements of UAV rotor blades can define the type of drone, while the propulsion turbine of a jet or the flapping wings of a bird can be statistically described by the radar m-D signature [44,45,46]. Another study [47] showed that distinguishing between a drone and a bird can be accomplished using machine learning algorithms by extracting features from m-D signatures. Several methods suggested the use of bistatic radar, where transmitter and receiver are not collocated, or multi-static radars in order to increase accuracy of UAV detection [48,49,50]. Compared to other technologies, radar is able to provide long-range detection up to several hundred kilometers, depending on the target radar cross-section (RCS). Its performance is almost unaffected by adverse light and overcast conditions [51]. On the other hand, challenges to the use of radar include the lack of automation and the high dependence on trained radar operators [52]. Moreover, radar is the most expensive equipment of all available drone detection sensors, and it requires national frequency spectrum licensing and environmental compatibility study.

In airports, radar sensors use large RCS in order to detect standard sizes of aircrafts moving with high velocities; thus, they cannot detect s-UAVs, which are small and slow-moving objects flying at low altitudes [53]. Radar sensors trace small targets at short, medium and long ranges; therefore, it is a common practice in aviation to use multiple radars with varying detection range in order to cover detection in an airport’s terminal area (TMA) and en route airspace [54,55]. Another drawback of radar sensors for tracking drones is the lack of geo-localization of GCS and the pilot of the invading UAV; thus, this surveillance technology is commonly used in combination with other detection sensors [55].

**Radio-Frequency Detection**

Radio-frequency (RF) scanners use passive detection technology and provide a cost-effective solution for detecting, tracking, and identifying UAVs based on their communication signature. They explore algorithms to scan known radio frequencies, so as to find and geolocate RF-emitting drones, despite weather and day/night conditions. Many studies have used RF scanners, either for locating a drone in space or for classifying FPV (first-persons view) channel transmissions [5,54,55,56,57,58,59,60,61,62,63]. Nguyen et al. [56,57,58] analyzed RF signals captured by software-defined radio (SDR) in order to detect commercial UAVs with high accuracy from a distance of up to 600 m. UAVs were traced with variable accuracy (64–89%), depending on the drone type. In [56], the signal strength patterns of Wi-Fi signals were analyzed from approaching and spying Wi-Fi drones. While the method uses a Wi-Fi receiver, its efficacy depends upon distance and a line of sight between the receiver and UAV. 

However, detection accuracy in environments with many Wi-Fi signals and among other emitting smart devices has not been validated. Scheller [59] investigated drone detection in heavy-RF environments, where an RF drone’s signature at a distance more than 100 m away could not be observed. Two studies [60,61] classified UAVs by using machine learning algorithms, while Peacock [61] proposed the analysis of UAV’s MAC address in order to detect and deactivate specific UAVs. Nevertheless, it is obvious that attackers can change a drone’s MAC address to avoid identification. Mototolea proposed commercial off-the-shelf FPGA based on SDR systems to detect and locate small drones [62]. Triangulation of drone’s position and its GCS is possible when using multiple RF scanners installed at an appropriate distance. Moreover, RF detection can provide early warning through the fact that the UAV and GCS transmit radio signals when the system is turned on. This allows enough time for the UAS to be detected, during launch preparation period, before taking off [62]. On the other hand, RF sensors cannot detect many drones at a time. Their accuracy is affected by other sources of potential interference, particularly due to line-of-sight obstacles [63]. Their effectiveness is valid as long as the UAS transmits a signal. However, malicious drones may fly autonomously, without emitting RF signals, in order to avoid RF detection or even transmit to a dedicated band that is not popular for FPV use.

**Acoustic Detection**

Drone propellers transmit an audio pattern that can be detected and used for drone positioning and classification by acoustic sensors. Usually, a microphone detects the sound made by a drone and calculates the location using the time difference of arrival (TDOA) technique, while more sets of microphone arrays can be used for rough triangulation of UAVs [64]. In most cases, acoustic sensors have a short detection range, less than 300 m [65]. They are subject to interference limitations with other audible noise, which is quite significant around airports. For UAV acoustic detection, researchers used microphone arrays with single board computers for performance evaluation of acoustic denoising algorithms [66]. Other researchers proposed drone detection frameworks by correlation [67] or using acoustic signature identification [68]. Acoustic fingerprint collection is a major issue for acoustic detection and identification; however, there are factors able to scatter sound waves, altering the direction of the sound, like for wind, temperature, time of day, obstacles, and other emitted sounds [68]. During a hot day with little wind in open plain areas, sound fingerprints will be significantly different than the ones during a cold, windy night in a forest [69]. Some researchers proposed methods able to triangulate sounds captured from centralized [64] and distributed [65] microphone arrays, in order to detect the location of low flying aircraft. Kim et al. [70] proposed a real-time drone detection system and used artificial neural network to increase the accuracy of classification. A background noise class was also introduced so as to separate the UAV sounds using the UrbanSound 8 K dataset [71]. Jeon et al. [72] proposed a binary classification model which uses audio data in order to detect UAV’s presence. Although acoustic sensors cannot be considered a primary detection source, they are often combined with other detection systems to enhance drone identification. Park et al. [73] proposed the combination of radar and audio sensors for identification of rotor-type UAVs by using neural network in their method.

Acoustic sensors can detect autonomous flying UAVs, with lower system costs and medium probability of detection with a higher false alarm rate (due to the increasing number of drone models), while geolocation of the operator is not provided [63]. Finally, acoustic sensors rely on a database of sounds emitted by known drones and might be deaf to drones not covered by the library. Algorithms can also identify the type of UAS and even differentiate between authorized and unauthorized UAS. However, in airport heavy-noise environments, where aircraft noise is enormous and overlapping, the use of acoustic sensors cannot be considered a reliable detection method.

**Visual Detection**

Imaging systems and cameras can be used both in the visual and infra-red spectrum to detect and classify drones. Not typically a primary detection source, electro-optical sensors use a visual signature to detect UAS, while infrared sensors use a heat signature. High-performance camera systems provide images as forensic evidence. They are often equipped with a high zoom capability to show small objects at a distance; however, they have range limitations [52]. Several researchers have suggested methods to detect a drone and its trajectory by using motion cues [74,75], visual marks [76], visual marks [76], and shape descriptors [77]. Neural networks and deep learning algorithms, when combined with optical data, can provide significant support and advanced intelligence to a UAV detection system, as presented in [78,79,80]. Rozantsev et al. [81] have used multiple fixed ground cameras for dynamics-based recovery of UAV trajectories. Opromolla et al. [82] used a vision-based approach for drone detection with the exploitation of template matching and normalized cross-correlation metrics. Gokcce et al. [83] employed vision-based drone detection and distance estimation using traditional features such as histogram of gradients (HOG). Researchers have also achieved detection and object classification by using hyperspectral images [84,85]. The methods can accurately locate and identify drones. However, since many similarities between the movements of drones and birds exist; there are high false positives on the one hand combined with high false negative rates on the other due to the increasing number of drone models and atmospheric opacity [54].

Thermal sensors use the non-visible electromagnetic spectrum and differentiate their operation from optical sensors. Thermal cameras can trace infrared radiation when emitted by flying objects in the form of heat. They use the long-infrared range of the electromagnetic spectrum, with a wavelength between 9–14 μm [43]. Several researchers have suggested using thermal cameras for UAV detection in obscure environments. Muller [86] proposed static short-wave infrared (SWIR) for night detection, increasing the UAV detection sensitivity in front of trees with waving leaves, with false alarm minimization. Birch and Woo [87] performed a comparison of drone detection at various distances using SWIR, mid-wave infrared (MWIR), and long-wave infrared (LWIR) imagers. In [88], a localization method was presented with 2D and 3D triangulation using images from multiple thermal cameras. Moreover, the use of thermal cameras offers the ability to visualize the surrounding environment with robustness, regardless of the external lighting and ambient conditions. When compared to optical RGB cameras, thermal cameras offer a tracing advantage with increased resilience against illumination changes [63]. On the downside, thermal cameras provide lower-resolution images and are more expensive than the electro-optical ones.

Finally, Church et al. [89] analyzed the detection of drones using a LiDAR sensor and found a good detection accuracy, within a range of a few hundred meters. Nonetheless, infrared cameras and LiDAR cannot identify drones because captured instances have rather low resolution [5]. Typically, in detection systems, a combination of cameras that capture visible and invisible wavelengths is applied to support observation throughout the day and night. It is hard for these to be used for detection alone; therefore, they are often paired with radar and RF options, as an additional tool for UAS detection, verification and forensics analysis.

**Comparison of Detection Technologies**

Based on a literature review of academic work on C-UAS we have summarized in Table 3 the advantages and drawbacks of detection sensing technologies. It is obvious that adopting a single sensor technology for UAV detection in airports cannot provide the desired situational awareness. Utilizing different sensors in a system is considered more efficient for drone detection systems, especially in the airport complex environment. Therefore, in airports, detection can be implemented in different ways, either as a distributed system on the airport perimeter or as a single point of detection capability. 

Taking into account the fact that UAVs may be requested to perform specific tasks in the airport premises, it is important to distinguish authorized UAV operations from misused drone flights [10]. The types of devices permitted to fly around airports should be identified, in as much detail as possible, with registration mark (if available), size, color, number of rotors, direction of travel, etc. Identifying the drone type and main characteristics, in the case of nefarious use, will provide the security team with information about the drone’s endurance time and which countermeasure is best suited for a response. 

In the near future, USA, Europe and other states are planning to develop and implement unmanned aerial system traffic management (UTM) systems and remote identification requirements for civilian drones, which will enable airspace authorities to segregate compliant and non-compliant drones [90]. UTM, as a traffic management ecosystem for UAV operations, will be separated from manned aviation ATM systems. However, services, roles and responsibilities, data exchange protocols, infrastructure, and performance requirements are under development for enabling the management of low-altitude uncontrolled drone operations. Since this initiative is under design, counter-drone technologies are essential for protecting airports from misused UAVs.

### 3.3. Mitigation Countermeasures

There are a number of technological solutions for mitigating threats from malicious UAS when approaching critical infrastructures. However, the adopted mitigation options should be legal, proportionate and properly risk-assessed. Two types of C-UAS technologies exist: electronic and kinetic. Electronic countermeasures can defeat UAVs by using communications link manipulation, RF jamming, or GPS spoofing. Kinetic interdiction refers to intercepting UAS by physical means. Both technologies are reviewed in order to examine their applicability in the airport environment. The analysis is resumed with a comparative table for the benefits and limitations of each mitigation technology.

**Electronic Interdiction**

Electronic or Signal Jamming is the intentional use of RF transmission, in order to block signals and disrupt communications between the GSC operator and the flying UAV. A radio-frequency jammer is a static, mobile, or handheld device, which transmits a large amount of RF energy towards the drone, masking the controller signal [54]. This results in the following reactions, depending on the drone’s design: (i) the drone makes a controlled landing in its current position; (ii) the drone returns to a user-set home location; (iii) the drone falls uncontrolled to the ground; and (iv) drone flies off in a random uncontrolled direction. Several studies have suggested disrupting incoming/outgoing communication for disabling UAVs [5,54,55,63,91,92,93,94,95,96,97,98,99,100,101,102]. Luo [91] applied radio jamming against a video link channel and showed that the FPV functionality was disabled, preventing the operator from maneuvering the drone. A jammer’s ability relies on the strength of its radio transmitter; however, the effective range cannot exceed a radius of a few kilometers [55].

Another option is GPS jamming, when UAVs use GPS navigation systems; however, mitigating a satellite-navigated drone is a much larger challenge than jamming an RF-controlled drone [63]. In order to effectively jam a satellite navigation signal, a new stronger signal is sent to the drone, replacing GPS communication, which the drone uses for navigation. Robinson [92] showed that applying GPS jamming to drones results in the vehicle drifting, increases the difficulty of controlling the drone, and prevents the return-to-home functionality from working. Mitch et al. [102] surveyed the signal properties of 18 commercially available GPS jammers based on experimental data and presents measurements of the attenuation of jamming efficiency. By dynamically altering the GPS coordinates in real-time, the drone’s position can be controlled and the drone can be directed to another landing zone.

Protocol manipulation refers to a third party which takes control of a UAS by impersonating its remote control. Signal instructions are emitted in order to confuse the UAS so that the manipulated signal is conceived as a legitimate one [54]. Many researchers have proposed methods to hijack and disorientate a UAV. They used replay attacks, which were applied from a malicious control station against weak uplinks of FPV channels [5]. Rodday [93] presented techniques for hijacking a USD 30k drone, by exploiting the XBee 868LP protocol and replaying commands of control which were sent from the GCS to UAV over the frequency of 868 MHz. Highnam et al. [94] showed that amateur drones, when using an MAVLink communication protocol (such as 3DR IRIS+, Erle-Copter etc), can also be hijacked using a replay attack. Davidson et al. [95] proposed a method to hijack a UAV by spoofing its downward camera. They directed a laser to the surface of the flying drone and influenced its stabilizing algorithm. Some studies [96,97,98,99,100] have demonstrated methods able to disable a drone with GPS spoofing while the UAV was moving in no-fly zones, thus preventing autonomous navigation towards its target. In order to take control of the UAS with a new communication link, protocol manipulation algorithms are enhanced with artificial intelligence. These manipulating signals offer a third party the opportunity to neutralize the rogue UAV by taking control of the flight, capturing the drone and downloading its data. However, this method may not be effective when command and control communications are encrypted, or when using a proprietary protected protocol [52].

**Kinetic Interdiction**

Many types of kinetic options are being proposed by researchers [5,103,104] and industry [52,53,54,105]. Their deployments have been tested mainly (i) on the battlefield in military missions; (ii) for the security of executive and government officials; (iii) in high-level special events. Such kinetic measures include the following:

- Net capturing is the attempt to physically capture a drone. An enforced and hardened UAV flies toward the intruding drone and carries attack nets in order to seize and bring back the targeted UAS. Such systems work at relatively short distances and are effective when the nefarious drone navigates with a low speed or does not maneuver.
- Birds of prey are trained birds with protective gear, which are used to attack and grab UAS, when entering into a restricted area. However, birds are also restricted and pose hazards when flying around airport areas due to possible conflicts with arriving or landing aircraft.
- High-power microwave (HPM) or laser fire: using high-power electromagnetic pulse or laser weapons, security teams are able to target and shoot down UAVs. HPM or high-energy lasers destroy electronic circuits and other vital segments of the drone’s airframe. It often causes UAVs to crash to the ground.

However, outside military use, kinetic techniques may not be a viable option, especially in crowded areas, due to the risk of the drone’s uncontrolled crashing or of triggering the deployment of CRBNE payloads. In most cases, they are not suitable for airports and the surrounding airspace, due to collateral hazards to aviation operations. Therefore, these kinetic interdiction measures may not be legal, depending on civil aviation rules.

Benefits and limitations of mitigation measures against misused drones are summarized in Table 4, where we can notice common drawbacks for all measures in their legitimate applicability to complicated airport environments.

In many countries worldwide, mitigation counter-drone systems are not allowed to be used in civilian environments, but only when applied by police and military operations [1,52,63]. There is some confusion and ambiguity regarding legal liabilities of C-UAS technology use, subject to numerous overlapping laws (such as aviation security laws, computer security and electromagnetic compatibility regulations). Adding to this ambiguity is the fact that most governments have not yet established comprehensive C-UAS-specific policies for protecting aviation assets, while airspace regulators continue to develop regulations for UAVs’ integration into commercial and civilian uses.

### 3.4. Counter-UAS Applied Techologies in Commercial Systems

In addition to academic research publications, in this subsection we have collected information about available C-UAS products and counter-drone technologies applied in commercial systems. Searching open-source databases for marketed counter-drone systems, we have investigated their technical characteristics and present a statistical analysis of sensor technologies used. There are at least 545 counter-drone products, based on open-source information, commercial publications and press releases [52,104,105]. C-UAS systems have been divided into three main categories, as presented in Table 5, where 178 systems (or 33%) have been designed only for detection purposes using a variety of detection sensors. The majority of C-UAS products, which are 218 systems (or 40%), provide mitigation technologies with interdicting UAV capabilities, while 149 systems (or 27%) are capable of both detection and mitigation.

**Figure 4:**
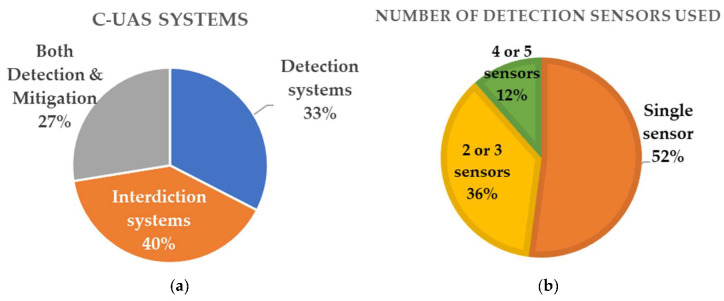
C_UAS systems: (**a**) Detection/mitigation Technologies Used. (**b**) Number of detection sensors.

Basing our analysis on C-UAS technical characteristics, in Figure 4a we have plotted the percentages of C-UAS systems which are capable of detection and mitigation, while in Figure 4b the numbers of sensors used for detection purposes in every system are exhibited. As shown in graph 4b, the majority of C-UAS systems (52%) use a single sensor for detection and the mainstream method is RF scanning, mainly due to cost–benefit advantages. More advanced and expensive systems use a combination of two or more sensor types (36%), usually combing primary surveillance methods with visual sensors. A minority of C-UAS systems (12%) employ a combination of 4–5 different sensor types, integrating RF sensors with visual cameras (both optical and infrared) and acoustic sensors.

**Figure 5:**
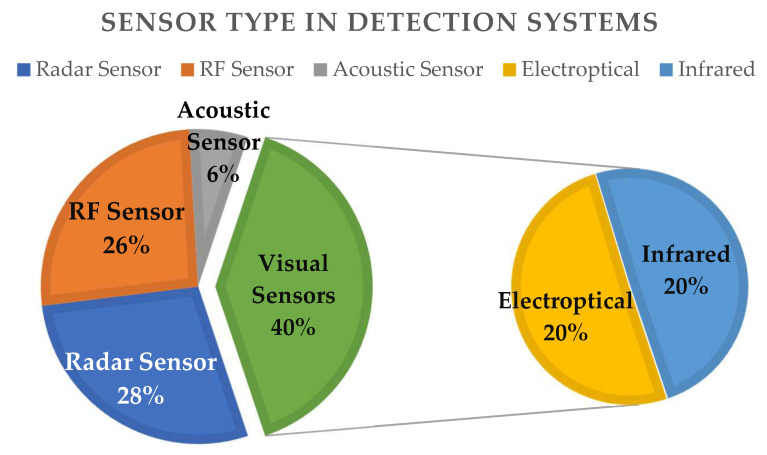
C_UAS systems: types of sensors used for detection.

Radio-frequency scanners and radars are the most commonly used detection elements (Figure 5). Radars are used in 159 (28%) systems, and RF in 147 (26%) ones. Visual systems are also popular with 40% of systems employing cameras for supporting RF detection. Electro-optical cameras and infrared systems, which are often used in conjunction, are equally applied in C-UAS systems, with a percentage of 20% each. Acoustic sensors are less common in use, with 6% application in products, mostly in conjunction with other detection technologies. This analysis is plotted in Figure 5.

**Figure 6:**
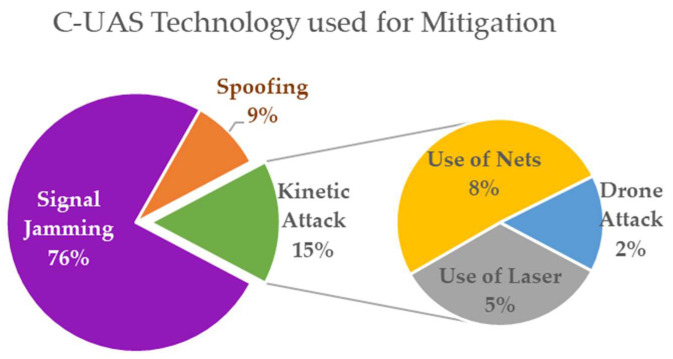
C_UAS systems: types of sensors used for mitigation purposes.

From the 367 available systems that have mitigation capabilities (either stand-alone or combined with detection sensors), 147 (or 40%) rely on a single mitigation technique, while 215 (or 58%) rely on two or more techniques. In Figure 6, types of sensors used for mitigation purposes are presented. RF and GNSS jamming techniques are counted distinctly, although they are often used in conjunction. Signal jamming (both RF and GNSS) is the most common interdiction method, with a percentage of 76% use in systems. Nine per cent of systems have spoofing capabilities, while kinetic methods are used in 15% of the systems examined. Among kinetic methods, 18 (or 8%) involve lasers, 27 (or 5%) employ nets, and 8 (or 2%) use a sacrificial UAV able to attack against intruding drones. Jammers are most commonly used for disabling drones. Some anti-drone jammers are directional RF transmitters in the form of mobile shooting guns that apply jamming to GPS signals and ISM bands, known to be used by drones (ISM bands are frequencies reserved internationally for industrial, scientific and medical purposes).

## 4. Attacks with Drones in Airport Critical Infrastructures: Scenario Analysis

Having examined the available sensor technologies and countermeasures for defending misused drones (both in industry and academia), in this section, we extend previous research on airport cybersecurity [106,107] and aviation cyber-resilience [108]. Therefore, in this work, we present and analyze various attack scenarios using s-UAVs (small drones) against airport facilities. Our main purpose is to exhibit the security and safety risks from misused drones and propose appropriate C-UAS and counteractions that are efficient and applicable in airports and support aviation resilience against airborne threats.

**Figure 7:**
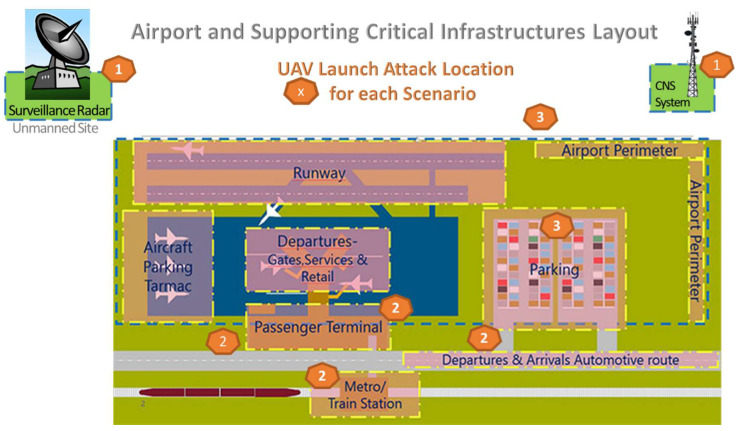
Typical airport layout and possible locations for launching a UAV attack on airport CI (each location spot number is connected with the number of the scenario presented).

In Figure 7, a typical airport layout is presented, which includes: (i) airport runways; (ii) aircraft parking areas; (iii) passenger terminal buildings; (iv) near-to-airport installations, supporting air traffic management; and (v) connecting public transport infrastructures. In this layout, we have marked with spot numbers {1, 2, 3} locations that are open to public and may be used as spots for launching UAV attacks. Each spot number is associated with the number of scenarios presented below. The following three categories of attack have been analyzed:

- Scenario (1): Drone attack to remotely located or unmanned sites near airports that support air traffic management (ATM) critical infrastructures;
- Scenario (2): UAS attack against airport wireless systems, information systems and data links;
- Scenario (3): Drone attack to ATM Systems, jeopardizing flight safety of manned aviation.

Each attack scenario is complemented by the following: (a) attack target background, which presents vulnerabilities and related research on similar attacks; (b) graphical representation of attack scenario; (c) attack analysis on a step-by-step basis; (d) impacted assets; and (e) impact evaluation with a resuming impact analysis table.

As we can notice in Figure 7, these three attack scenarios may be launched from different spot locations (inside and outside airport premises), which are: (a) near or inside the passenger terminal area; (b) in the parking area; (c) near public transport connections (bus/metro/train station); (d) near or outside the airport perimeter; and (e) in peripheral ATM sites, which are located outside the airport perimeter. These eight spots are public locations accessible to all, often overcrowded and often with less strict security measures. As a result, the scenario analysis presented below, as escalated on a step-by-step basis, covers almost all possible attacks that can be performed by malicious actors, exploiting UAS capabilities inside and around airport facilities.

### 4.1. Scenario 1: Drone Attack to Unmanned Sites, Supporting ATM CIs

Attack Target Background: spying aeronautical telecommunication systems for vulnerabilities and information gathering are the first step for target reconnaissance when preparing a malicious attack. A drone’s FPV channel provides an excellent tool for a malicious operator to spy on any target without being detected since the operator can maneuver the drone and collect information from miles away. The UAV’s attached camera can capture data, obtain high-quality pictures, record video and send back information gathered about vulnerabilities scanned in order to prepare a successful attack. Several studies have shown that drones equipped with radio transceivers can be used for extracting unencrypted information from radio transmissions or even create RF noise or telecommunication interference [5,12,106]. Besides, drones can cause significant damage to unmanned sites that support aeronautical telecommunication systems by carrying explosive payloads and even by self-exploding, while targeting antennas, navigational aids and other critical infrastructure. Such incidents have already occurred in the past, in many airports, like in Saudi Arabia [109] and the Middle East [110], causing serious fatalities.

Graphical Attack Representation

**Figure 8:**
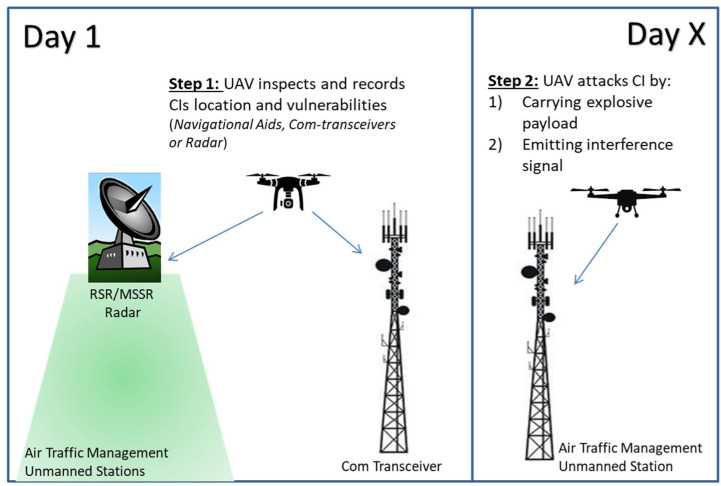
Drone attack to unmanned air traffic management (ATM) CIs.

Graphical attack representation of drone attack to unmanned air traffic management (ATM) CIs is shown in Figure 8

Attack Scenario Analysis

Step 1: A UAV driven by a malicious user can perform a reconnaissance flight above and nearby aeronautical telecommunication systems in order to monitor and record site vulnerabilities, with the intent to prepare an attack after a period of time. As shown in Figure 8, the drone can be equipped either with a video camera, optical sensors with night vision capabilities (for after dark flights), or a radio-frequency (RF) analyzer to detect wireless communications and RF signals.

Step 2: If step 1 is successful, as presented in left-hand part of Figure 8, the drone may return to the site, as shown in the right side of Figure 8. After a period of time, having elaborated all information gathered about the attack target, the UAV can realize (i) a physical attack by carrying either an explosive payload against the physical integrity of the CI facility or (ii) a cyber-attack by using RF jamming equipment to interfere with existing ATM communication systems.

Impacted Assets: Communication, surveillance and navigation (CNS) systems are often unmanned sites, far away from an airport’s main establishment. These systems include (a) aeronautical telecommunication systems; (b) navigational aids, which provide guidance, location, and direction to airplanes; (c) surveillance systems (primary and secondary surveillance radars), which detect and report the position of aircrafts for air traffic control purposes [111]. Very-high-frequency omnidirectional ranges (VOR) are often located nearby airports and provide a bearing to and from the station, along with magnetic direction. Non-directional beacons (NDB) broadcast a signal on an AM frequency to support the pilot’s direction and orientation [111]. In addition to CNS, supporting equipment may be affected, like power supply and HVAC (heating ventilation air-conditioning) stations, where stopping or downgrading their operation may create cascading operational problems to main CNS systems and airport CI operation. All of the above assets, which are often located in distant areas from airport security systems (in unmanned sites), may be vulnerable to aerial attacks.

Impact Evaluation: CNS systems are exposed and, when assaulted by a malicious UAV, may lose integrity and their operational efficiency. This results in ATM service degradation and traffic flow slowdown for safety precautions. Moreover, airspace capacity limitations, flight delays or cancelations may also occur. Economic losses to air navigation service providers and aircraft operators supervene, due to downtime for repair and integrity checks. There can also be material or service loss and additional legal liability to air navigation providers and/or airport facilities. Table 6 presents an impact evaluation for each step of the attack scenarios analyzed. This table includes impacted assets and a description of areas impacted (economic, legal, reputation, human or material/service loss), along with information security impacts affecting confidentiality and/or integrity and/or availability.

### 4.2. Scenario 2: UAS attack on an Airport’s Wireless Network and IT Infrastructures

Attack Target Background: An airport operations center, supported by a central information network, connects airport facilities and serves as an interaction point for all airport community stakeholders. It manages processes from airside and flight control systems to landside operations and ground handling systems [112]. As a result, in modern airports, the operations center acts as the central point of command, effectively managing all data interchange and information sharing. Information is extracted from a series of sensors and smart devices and communicated though wireless LANs (802.11) or wide-area wireless networks (WiMax, Lorawan) using ground-based line-of-sight data-links, due to expanded airport borders. Drones equipped with wireless antennas and software can take advantage of access point communications and can sniff and capture data packages sent between wireless connected devices. Recent research [113,114,115] has proven that drones can be used in order to monitor an access point, capture the communication packets and record detailed network information. According to Gittleson [115], malicious software can be installed on a drone (called Snoopy) to harvest personal information and to track and profile smartphone users. Snoopy can also sniff RFID, Bluetooth, and IEEE 802.15. A Snoopy drone can exploit the WiFi, impersonate the identified network and trick smart devices into joining it, so as to collect all the information entered on this disguised network. In addition, UAVs can perform 3D through-wall mapping [116], leak data from air-gap computers [117], or even carry traditional spying devices to eavesdrop on conversations [5]. With the use of RFID tags, attack targets can be traced by a RFID reader at distances varying from one up to a few hundred meters [118]. As a result, UAVs equipped with RFID readers can trace RFID tags, navigate and locate themselves via specific points and identify attack targets [119,120]. All the above attacks to wireless networks can be performed by a sUAS in an airport environment, which is overcrowded with passengers, airport community employees and various commercial activities and full of wireless communications and smart applications. 

Graphical Attack Representation

**Figure 9:**
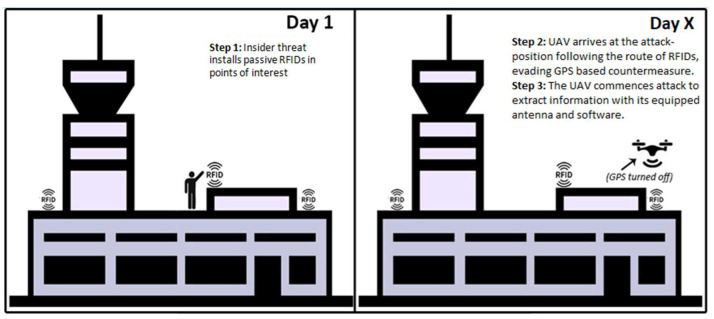
Drone attack in airport facilities assisted by an insider.

Graphical attack representation of drone attack in airport facilities assisted by an insider is shown in Figure 9.

Attack Scenario Analysis

Step 1: An insider takes advantage of free entrance on the rooftop of the building and/or nearby facilities and infrastructure without being noticed by security controls. He is able to distribute RFID tags in order to mark sensitive locations, e.g., airport server rooms, wireless routers, arrays of integrated smart sensors and security camera networks. This step is presented on the left-hand part of Figure 9, followed by second step on the right-hand part.

Step 2: A mini-UAV performs an attack some days later, targeting distributed RFID tags. Assuming that the airport has countermeasures against drones, and in order to avoid geofencing, the UAV turns off its GPS navigation system and follows the route identified by the distributed RFID tags towards its attack position. As a result, the drone requires less energy to be guided to its destination relative to a GPS-navigation-based drone, so this expands its flight endurance time. Its small size and low-altitude flight can make the UAV untraceable for ground surveillance radars, while anti-drone protection based on GPS-spoofing cannot affect its route towards the attack target. If the flight is performed during nighttime, also it can be untraceable by optical security sensors and security patrols.

Step 3: If not traced, the UAV identifies its target and, while equipped with a wireless antenna and supportive software, it can take advantage of vulnerable access point communication. As a result, it can sniff and capture the packages sent between Wi-Fi-connected devices and wireless sensors, extract information and send it back to its malicious center of command. Likewise, a drone can perform an acoustic attack to capture and record voice communications and reveal sensitive information. Even worse, it can also perform a physical attack by carrying an explosive payload.

Impacted Assets: Server rooms, Wi-Fi routers and smart sensors used for airport operations monitoring and airport data center security may be put in jeopardy. Passenger handling systems, automated vehicle identification, and RFID-based asset tracking systems may be impacted. Communication confidentiality, including ground-based line of sight data-links and ATM signals, may be compromised.

Impact Evaluation: An information, communication and surveillance system can be disrupted, downgrading airport services. An airport’s operation can be disorganized or forced to close certain services if a data leak is detected. Passengers and employees’ personal information can be disclosed or stolen, leading to legal liabilities, economic fines and reputation loss. Airport sensitive corporate information may be exposed to malicious actors. Airport operations will be forced to stop operations for safety reasons if a UAV is detected in restricted airspace areas. Table 7 presents an impact analysis for each step of an attack in scenario 2 and the impacted assets.

### 4.3. Scenario 3: Cyber-Physical Attack to Air Traffic Management Systems and Manned Aircrafts

Attack Target Background: ADS-B (automatic dependent surveillance—broadcast) systems are an emerging surveillance technology, recently introduced in aircraft navigation as the cornerstone of airspace management modernization [121]. An ADS-B transponder periodically broadcasts information about an aircraft’s current position and enables it to be tracked by surveillance systems. The information can be received by ATC ground stations and by other aircraft in order to provide situational awareness, allowing self-separation and supporting traffic collision avoidance systems (TCAS).

However, according to many researchers, a plethora of active attack scenarios and serious security breaches have been presented, posing a risk to the integrity of surveillance systems [122,123,124]. The system is susceptible to hacking, where attacks may range from passive actions (eavesdropping) to active attacks with the use of malicious drones. ADS-B and ATM radio technologies are broadcasted unencrypted. As a result, a well-equipped attacker can receive and send messages or overshadow existing signals. In addition, due to the weak security posture of satellite communications, hundreds of in-flight aircrafts are accessible and vulnerable to message jamming, replaying injection and other active attacks [124]. 

Graphical Attack Representation

**Figure 10:**
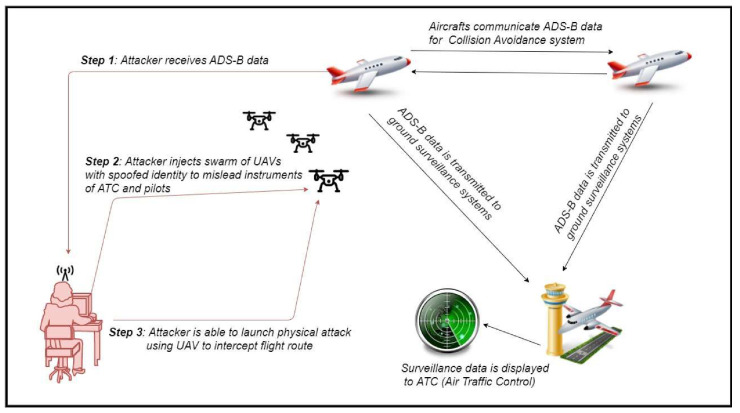
Communication attack on ATM systems.

Graphical attack representation of communication attack on ATM systems is shown in Figure 10.

Attack Scenario Analysis

Step 1: An attacker is located near the airport facility and is equipped with software-defined radio supported by an ADS-B receiver/transmitter chain with GNU radio. Based on this equipment, he receives ADS-B data from passing aircraft. As a result, the malicious user is able to collect air traffic information about aircraft traversing the area and transmitting their position (aircraft type, identification mark, position coordinates, altitude, speed, direction, destination, etc.), in order to prepare his attack.

Step 2: The attacker launches into a designated airspace a single UAV or a swarm of drones equipped with ADS-B transponders, as shown in Figure 10. Their identity is spoofed based on data collected in step 1 (replay attack). Hence, malicious drones transmit false ADS-B data, pretending to be commercial aircraft. As a result, confusion is created in the airport’s surveillance system and ATM, which forces air traffic control to stop or slow-down air traffic in the impacted area for safety reasons.

Step 3: Even worse, the attacker may escalate by launching a physical attack against approaching aircraft, since data collection about the airplane’s position, destination and technical data enable him to calculate the airplane’s position in future time, to target and send a UAV towards this aircraft. This midair collision hazard can cause safety issues, with serious fatalities and damage to both aircraft, especially during take-off or landing, as already presented in [13].

Impacted Assets: Air traffic control, secondary surveillance systems (ADS-B data), aircraft safety during take-off and landing phases, aircraft safety, and separation minima can be violated. The airport’s infrastructure and manned aircrafts may be seriously damaged by uncontrolled UAV flights and crashes, in case of mid-air collision hazards. Last but not least, human losses or injuries are also intolerable.

Impact Evaluation: Surveillance integrity is threatened, and air traffic can be disrupted or downgraded for safety reasons. ATC operations should immediately close violated airspace. Aircraft safety is jeopardized; standard routes may be deviated, flights cancelled and air traffic diverted to other airports. A serious accident with an aircraft may cause fatalities, serious material loss and destruction of an airport’s CIs. The airport will close for further incident/accident investigations with serious economic losses and a negative reputation. Table 8 presents the impact evaluation for each step of an attack in scenario 3, along with impacted area analysis.

## 5. Proposed Countermeasures for Airports

Airports may differ in size, design layout, air traffic flow and capacity, proximity to populated areas, etc. However, some hazards associated with UAVs are common to all airports, bypass standard security measures, and should be addressed with priority while examining UAV integration in airport operations. In civilian airspace, drones are not yet required to carry transponders, so they cannot be detected and tracked with existing air traffic control systems. Relying on visual observation to detect drones is equally ineffective, since s-UAVs can become invisible to the naked eye. Although detection methodologies for tracking drones have been developed, the small size of drones and the varieties of design and material used in UAVs pose challenges to detection systems, as already described in Section 3. In this section, we propose countermeasures for preventing, detecting and defending misused drones from invading airport premises. For each scenario, a proposed C-UAS protection plan is designed and graphically presented, aiming to increase airports’ resilience and robustness.

### 5.1. Scenario 1: Drone Attack to Unmanned Sites Supporting ATM-Critical Infrastructure

**Figure 11:**
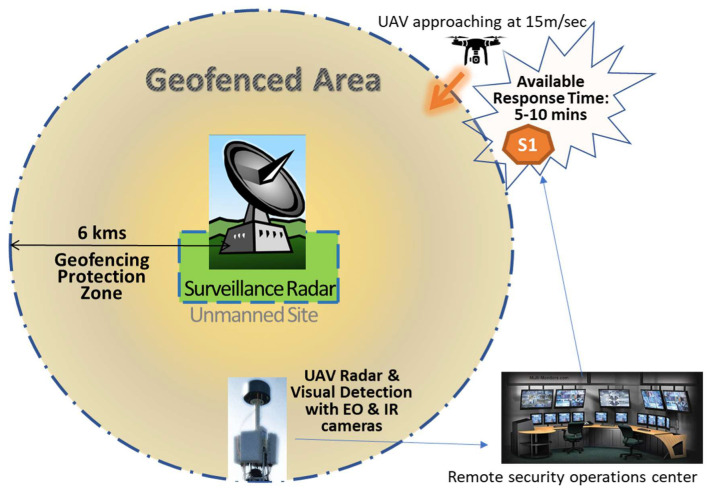
C-UAS protection plan for scenario 1.

CI vulnerability is higher when an asset is remotely located in unmanned sites. In the first scenario, with inadequately secured ATM sites, it is important to establish geofencing barriers as a standard preventative measure. Due to the lack of physical protection measures from airborne threats, enhanced safety geofencing zones of 6–10 km around ATM sites are required. These should be designed, legislated and communicated to all aviation stakeholders and the UAV industry while being integrated into publicly available aeronautical information packages (AIP). In addition, a sensor detection system must be installed, able to track, identify and locate any incoming drone, which may overpass geofencing areas. A wide-area surveillance radar or RF detectors are proposed as the primary detection method. Moreover, secondary sensors, such as electro-optical or infrared cameras, may support the detection system and confirm the type of intruding UAV, while also providing additional information about its payload. For example, a PTZ (Pan-Tilt–Zoom) camera may be able to show whether a drone appears to be carrying explosives or simply a video camera for information gathering and record keeping. Installation of video surveillance systems, with remote monitoring connections to an airport’s security operations center, is also proposed, instead of enhanced physical protection of unmanned sites with security guards. However, security operators may only have a limited window of time to make decisions and take countermeasures when an incoming drone is considered malicious. In our case, assuming that (a) the incoming drone is travelling with an average speed of 10–20 m/s and (b) a geofencing perimeter of 6 km exists, the available response time is between 5 and 10 min, from invasion time into the restricted zone, before the target is reached. The proposed C-UAS protection plan for scenario 1 is graphically presented in Figure 11.

Finally, regular integrity checks and calibration of ATM equipment performance, backup and redundancy design for critical assets, and contingency operational plans for ATM services are also effective resilience measures. These will enhance the integrity and availability of aviation services in case of emergency or failure and prevent air navigation service providers (ANSPs) from service degradation while under attack.

### 5.2. Scenario 2: UAS attack on an Airport’s Wireless Network and IT Infrastructure

In the aviation context and according to national and international regulations, almost every airport has appropriate airspace limitations and prohibited areas according to state aeronautical information packages (AIP). These restricted areas are usually protected by a geofencing shell, acting as a virtual security shield that keeps drones away when their navigation is based on programmed GPS data. In our scenario, since the malicious actor has turned off GPS to avoid geofencing, only detection sensors can protect the airport from such nefarious drone flights. Surface surveillance radar systems on airport premises should be able to track sUAS moving slowly or hovering over airport critical infrastructure. When systems are supported by secondary sensors, such as electro-optical or infrared cameras, C-UAS can monitor and record any unauthorized UAV flight. They can also provide additional information about a drone’s payload. These detection sensors should be distributed around the airport perimeter, especially covering public access areas, based on security experts’ risk assessments. However, it is more important to be able to locate and capture the UAV pilot than chase any flying object while approaching airport facilities. Therefore, security guards should be adequately trained in order to perform regular security patrols, carry mobile RF scanners and be able to forestall any UAV illegal flight, even before it starts. Some kinetic countermeasures could also be applied by security trained personnel, (e.g., launching net capturing drones or using directed RF jammers), provided they are compatible with aviation rules and authorized by civil aviation.

**Figure 12:**
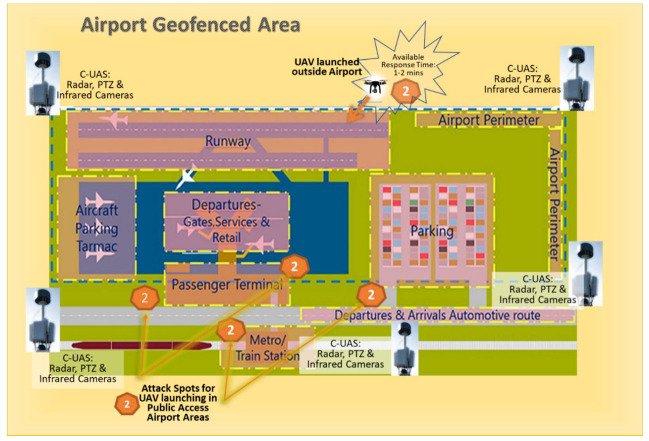
C-UAS protection plan for scenario 2.

The potential of insider threats exposing airport vulnerabilities should be eliminated. The airport community and employees should be well informed and discouraged with appropriate measures. They have to be alerted, act as spotters and inform security agencies in case of any suspicious drone sighting. In addition, closed-circuit television systems (CCTV) for 24 h surveillance will discourage malicious insiders from mis-performing or considering any action opposed to airport security. The proposed C-UAS protection plan for scenario 2, with detection sensors distributed around the airport perimeter and close to publicly accessible areas, is graphically presented in Figure 12.

### 5.3. Scenario 3: Cyber-Physical Attack to Air Traffic Management Systems and Manned Aircrafts

**Figure 13:**
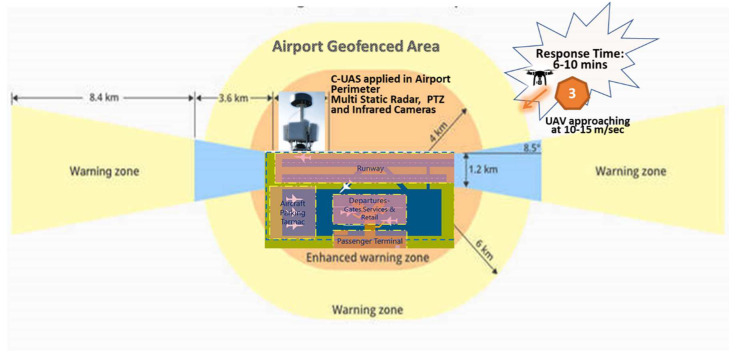
C-UAS protection plan for scenario 3.

Terminal airspace is required to be adequately protected and cleared by mitigating any airborne hazard. It is obvious that uncontrolled flying, landing or crashing of UAVs into obstacles or ground is an unacceptable situation in the aviation context. If an attack is launched outside the airport perimeter, the response time may vary in minutes depending on the launching spot distance and average UAV navigation speed. However, in case the attack is launched inside airport premises, the available response time is only few seconds, so it is vital for security teams to immediately react and prevent such actions, as proposed in scenario 2. Enhanced geofencing, using a 3-dimensional bow-tie shape to create virtual fences according to an airport’s risk management guidance, is an effective preventing measure. Thus, a geofencing warning zone expands the available response window for both ATM controllers and security guards, to detect, identify and react to incoming misused drones from a few seconds to few minutes, as presented in Figure 13.

Moreover, detection measures, including surface surveillance radar, RF detectors and visual sensors should be expanded in the vicinity of the airport, provided that they are compatible with aviation rules and are legally authorized. Air traffic control should receive data validated and multilaterated by surveillance radar systems so as to be able to distinguish malicious drones from airplanes, if their identity is spoofed. Confidentiality and authenticity features in traffic data should be enhanced with cryptographic protection for ADS-B systems. ANSPs must upgrade the surveillance technologies used by the current network of primary and secondary radars, in order to support and complement ADS-B technology.

Last, but not least, public awareness with safety promotion campaigns for UAS no-fly zones, along with educational leaflets and advertisements, will minimize uninformed or ignorant drone enthusiasts flying UAVs in the vicinity of airports. Drone registration, remote pilot training and licensing requirements are crucial elements that need to be specified, with clear financial and legal consequences for violating aviation rules and intruding into restricted airspace.

## 6. Discussion on C-UAS Applicability in Airports and Resilience Plans

Although there are a number of technological C-UAS solutions, as discussed in previous sections, no international standards exist for the proper design and use of C-UAS systems in airports and their critical infrastructures. The applicability of UAV-interdicting measures remains an open challenge to the complicated airport environment. According to the FAA, airports seeking to deploy UAS detection systems should be aware of the deployment hazards of such systems since they may implicate provisions of law, even when C-UAS are marketed as passive detection systems [125].

Terminal airspace should be adequately protected; however, the risk of interference with legitimate communications is a serious concern. Especially in the airport approach area, it is vital to eliminate any interfere with other important radio signals for aviation, such as instrument landing systems (ILS), surveillance approach radars, radio communications, etc. Moreover, RF jamming for civil use is illegal in many countries worldwide (EU, Canada, USA, Australia), and, as such, jamming cannot be used as a mitigation option in many airports. Likewise, jamming GPS/GLONASS signals near an airport is also considered dangerous for civil aviation, since many airplanes nowadays rely heavily on satellite navigation for take-off and landing procedures.

Airfield operators must remain within the law when using C-UAS technologies. The risks to the wider community should be fully assessed and understood. A clear decision-making process should be established to allow the airport operator to make the most appropriate decisions based on solid and accurate information. As exhibited in the previous sections, in most cases, aggressive mitigation measures cannot be implemented in civilian airports due to existing aviation laws and legal restrictions. Therefore, it is recommended for airport operators to establish coordination channels with security agencies, such as the police, the military and the Civil Aviation Authority, in order to strengthen their defense capabilities and ensure a more joined-up response. In case a drone falls within the airfield boundaries, the operator should also consult the police and legal authorities before approaching it, as it may contain vital forensic and digital evidence that could be used for investigation and legal prosecution.

Aiming to enhance airport resilience and robustness in confronting UAV attacks, aviation stakeholders have to develop efficient contingency plans with clear safety and security measures to protect their critical assets. While planning their strategy for increased resilience and robustness, airport operators have to consider doing the following:

(i) Implement an effective UAS detection system and create an internal reporting point for drone sightings. It is imperative to understand which part of a facility’s airspace has been infringed upon and locate the drone at all times during the incursion.
(ii) Identify the drone and understand the type of UAV being used, what threat may be posed to the airport operator or airspace management and what mitigation options are available.
(iii) If any mitigation options are adopted, they must be legal, proportionate and properly risk assessed, so as not to create any other hazard to the wider airport community.
(iv) An appropriate liaison with security partners and legal agencies (police, civil protection and civil aviation authorities etc.) should be established, in order to coordinate the response when an incident takes place.
(v) Whenever a drone interrupts an airport’s operation, and before resuming the flight schedule, the operator should confirm that the airspace is clear, the drone is disabled and it is safe for operations to restart.
(vi) Ensure that the business continuity plan, developed for airport operations, has included such types of UAV disruptions, while regularly exercising a preparedness scenario involving all aviation stakeholders.

## 7. Conclusions

Ranging from insect-sized to several tons in weight, drones are extremely versatile and can perform a large variety of tasks, transforming civil protection, security patrols, asset delivery and commercial and entertaining activities. Among the advantages of commercial drones are their relatively low cost, easy reach, great work productivity and capacity to reduce risk to human life. These features have led to their mass commercialization. Nevertheless, regulation and oversight remain immature, particularly regarding dual use of civil drones that can be easily turned into armed drones or weaponized for criminal purposes.

Drone-related incidents at critical infrastructures, including airport facilities, are expected to rapidly proliferate in frequency, complexity and severity as drones become larger and more powerful. The use of drones can appeal to nefarious actors since they are relatively inexpensive and provide the means to attack a target with low risk to the perpetrators. Critical infrastructures need to be protected from such aerial attacks through effective vulnerability assessment, risk management and resilience actions.

Although airport environments are complicated with a variety of sizes and design features, they have similar security requirements for protecting their facilities, detecting and identifying misused drones and taking effective countermeasures. Based on an extensive literature survey on C-UAS technologies, we have developed three categories of attack scenarios in airport premises and proposed an efficient C-UAS protection plan for each case. Geofencing as preventing measure and a variety of detection sensors can be implemented in different ways, depending on risk appetite, either as a distributed system on the airport perimeter or as a single point detection capability. Multiple radars with different detection ranges provide the necessary primary surveillance method in airports. Since it is important to identify the types and payloads of invading drones, we proposed a combination of radio-frequency sensors with visual detection sensors (electro-optical and infrared cameras), which can provide supplementary surveillance around an airport’s extended perimeter.

However, defending airports against unwanted drone activity is a wide and deep problem set. Despite the variety of technological mitigation solutions available, airfield operators must remain within the law when using disruptive technologies, and the risks to the wider community should be fully assessed and understood. A clear decision-making process should be in place to allow the airport operator to make the most appropriate decision based on solid and accurate information. Clearly, safety is the priority within the aviation context. Any decisions against a flying object should be appropriate, proportionate and necessary, with documentation and a rationale for making them.

Furthermore, appropriate responses taken by operators before, during and immediately after any UAV incident should be developed in a contingency action plan to minimize the impact on key stakeholders. Airports should rely on support and co-ordination from official security services, the military and industry partners to increase resilience and robustness.

C-UAS technology poses a wide range of practical, legal, and policy challenges in airport’s environment. A lack of common standards in the C-UAS industry means that there is a wide variance in the effectiveness and reliability of available systems. Efforts to identify new methods that will protect airspace and coordinate manned with unmanned aviation are ongoing, with unmanned aerial system traffic management (UTM) systems and remote identification requirements for civilian drones being designed. After all, further development of civilian and commercial UAVs and their integration into evolving smart cities is dependent on the ability of drones to operate in various areas of the airspace, especially at very low levels, without posing any risks to safety, security or privacy within society and its critical infrastructures.

## Figures and Tables

**Table 1:** UAV classification based on weight, altitude, range and payload.

Category	NASA UAS Class	Weight (in kg)	Normal Operating Altitude (in m)	Mission Radius, Range (in Km)	Typical Endurance (in hrs)	Payload (in kg)	Available UAV Models in Market
Micro	sUAS Class I	<2	<140	5	<1	<1	DJI Spark, DJI Mavic, Parrot Bebop2
Mini		2–25	<1000	25	2–8	<10	DJI Matrice600, DJI Inspire2, Airborne Vanguard
Small		25–150	<1700	50	4–12	<50	AAI Shadow 200, Scorpion 3 Hoverbike
Medium	Class II	150–600	<3300	200–500	8–20	<200	Griff 300, Ehang 216
Large/Tactical	Class III	>600	>3300	>1000	>20	>200	Boeing X-45A UCAV

**Table 2:** Number of new publications with the term “C-UAS” based on a Google scholar search.

Year	2014	2015	2016	2017	2018	2019
Num. of Scientific Publications	99	124	134	182	178	234

**Table 3:** Comparing C-UAS detection technologies.

Method	Benefits	Limitations
Radar	Long-range primary surveillance detection system up to 100 km, depending on RCS and altitude	Detection range dependent on drone size and radar cross-section (RCS)
		Radar systems designed for manned aviation cannot detect small flying objects
	Can track most drone types, regardless of autonomous flight	High acquisition and installation cost
	When combined with machine learning algorithms, can distinguish birds from drones.	Requires a transmission license and frequency check to prevent interference with other RF transmissions
	High-accuracy tracking while in angle range of observation	Hard to detect low-altitude-flying, slow-moving or hovering UAVs
	Able to track multiple targets simultaneously when using multi- tracking coverage	No pilot tracking capability or ground control geolocation
	Bistatic and multi-static radars increase accuracy of UAV detection	Lack of automation and high dependence on trained radar operators
	Independent of visual conditions (day, night, overcast weather, etc.)	False positives with similarly shaped objects (birds, clouds, etc.)
	No need for RF or acoustic signal	Environmental compatibility study is needed
RF detection	Lower cost than radar sensors with a medium range up to 600 m	RF signal required, cannot detect autonomous flying drones
	Detects certain radio frequency bands where UAVs and GCS communicate for command and control (C2)	Electromagnetic interference and loss of sight degrades detection capabilities
	Can capture RF emitted by UAVs and is able to locate UAVs and controllers	Variable detection accuracy depending on drone type and frequency band
	Can capture WiFi-emitting drones	Attacker can spoof MAC addresses
	High-accurancy detection	Can detect only a few UAVs at a time
	Early warning capability even before UAV takes-off (when turned on)	Less effective in heavy-RF environments with a range less than 100 m
	Triangulation is possible with multiple RF sensors	Detection limitations for swarm of drones
	Machine learning algorithms can classify drone transmissions	Some passive systems may emit RF signals, despite being characterized as passive systems
	Passive detection, no license required	
Acoustic	Classification based on acoustic signature	Depends on an available library of already-captured sound signatures
	Can differentiate between authorized and unauthorized UAS	Higher false positives due to the increasing number of drone models
	No need for RF signal for detection. Can detect autonomous flying UAVs	Unreliable detection at range >300 m
	UAV detection can extend beyond line of sight	Does not work as well in noisy environments
	Classification based on UAVs’ acoustic signatures	Detection limitations for swarms of drones
	Time difference of arrival (TDOA) technique is used for UAV localization while triangulation is possible with an array of distributed sensors	Detection performance is affected by wind direction, temperature, line of sight and signal reflections due to obstacles
	Low-cost sensors	Not used as a primary detection source
	Can provide drone direction or rough estimation	No pilot tracking capability or ground control geolocation
Visual	Detects visual signature for electro-optical (EO) cameras to classify UAVs	Need for human interference or artificial intelligence to efficiently detect UAVs
	Detects heat signature infrared spectrum for thermal (IR) cameras	Not used as a primary detection source (both EO and IR cameras)
	Can distinguish drones from birds, especially with IR sensors	Both have detection limitations based on resolution capabilities Hard to capture swarms of drones
	No need for an RF signal emitted by UAVs to capture	IR and EO cameras need a direct line of sight to detect UAVs
	IR cameras visualize surrounding environments, regardless of the external lighting or weather conditions and even in total darkness	EO Cameras depend on daylight and outdoor illuminance conditions (overcast, darkness, etc.)
	Can record sightings and use for further investigation	May confuse UAV with a bird or similarly shaped small airplane
	Can record incidents as forensic evidence for legal actions	Range limitations depending on weather conditions (clouds, rain, fog, mist, etc.)

**Table 4:** Comparing C-UAS mitigation measures.

Method		Benefits	Limitations
Electronic Interdiction/Signal Jamming	RF Jamming	Use RF transmission to block signals and disrupt C2 between the GSC operator and UAV	RF interference in crowded RF areas. May also jam and interrupt other communication signals
		Medium range up to a few kilometers, depending on emitting power	Cannot affect autonomous driven drones (without an active RF link)
		Static, mobile, or handheld device	Illegal use in many countries
		Programmable based on RF sensor scanning	May cause uncontrolled UAV flights and crashes
		Disrupts radio-frequency (RF) communication link sCan include WiFi links	Needs special licensing for approved use, based on electromagnetic compatibility regulations
		Use of directional jamming to minimize interfering	A jammer’s ability relies on the strength of its radio transmitter
	GPS Jamming	Replaces GPS communication, increases difficulty to control the drone	Cannot work if UAVs disable GPS or use encrypted GPS (military mission)
		Medium to short range, depending on satellite constellation	Dangerous when used near airports, because airplanes also use satellite navigation
		Disrupts Global Positioning Satellite communication link	Illegal procedure in many countries. Needs special licensing for approved use
		Prevents the return-to-home functionality	May cause uncontrolled UAV flights and crashes
	Protocol Manupulation	Replaces the communication link and takes control of drone operation	Illegal procedure for civilian use, acts against computer fraud and abuse
		Employs algorithms enhanced with artificial intelligence	Not always successful, especially when encryption is used for C2 links
		Can drive a malicious UAV to a designated area	Complicated method, not always successful
		Low-cost technique, based on attackers’ ability	Cannot affect autonomous driven UAVs not using GPS
Kinetic Physical	UAV Net Capturing or Birds of Prey	Active and aggressive countermeasures	May cause collateral fatalities to other aircrafts. Not appropriate for airports
		Net capturing: enforced and hardened UAVs physically capture a drone	Net capturing efficiency depends on UAVs’ flight behavior, reaction time etc.
		Birds of prey are used to attack and grab UAS	Birds also pose hazards when flying around airports
		Captures and drives UAVs in a specific area.	Depends on speed or maneuvering capabilities of rogue UAVs
Kinetic Electronic	High Power Microwave or Laser Guns	Aggressive and long-range countermeasures	Can have negative effects on other passing aircrafts with fatal consequences
		Destroys electronic systems of UAVs	May cause uncontrolled UAV flights and crashes
		Disables drone flight	Illegal in civil aviation contexts. Violates aviation security laws

**Table 5:** C-UAS Products available in the market or under development.

Number of C-UAS Products	545	%
Systems Capable of Detection	178	33%
Systems Capable of Mitigation (Interdiction)	218	40%
Systems Capable of Both Detection and Mitigation	149	27%

**Table 6:** Impact analysis of scenario 1.

Threat/Hazard	Impacted Assets	Impact Analysis		
		Description and Impact Areas (*)		on CIA (**)
Spying aeronautical CNS systems for vulnerabilities and information gathering	Air traffic management, communication, surveillance and navigation (CNS) systems, such as: - Navigational aids (VOR, NDB, DME) - Surveillance systems (RSR, MSSR) - Aeronautical telecommunication systems. - Power supply and HVAC remote stations	Site and system vulnerabilities exposure	R, L	C
Drone equipped with a radio-frequency (RF) analyzer detects wireless communication and RF signals.		RF signals exposure	R, L	C
UAVs carry RF jamming equipment to interfere with existing RF signals’ communication systems		CI operation interference, signal jamming and communication loss. Airspace capacity limitations	E, M, R, L	I, A
UAVs carry explosive payloads against physical integrity of CI facilities		CI physical damage. Loss of operational efficiency. Air traffic flow slowdown for safety precautions. Human injuries	E, H, M, R, L	I, A

(*) Impact areas: E = economic, H = human, M = material/service loss, R = reputation, L = legal. (**) Impact on information. CIA: C = confidentiality, I = integrity, A = availability.

**Table 7:** Impact analysis of scenario 2.

Threat/Hazard	Impacted Assets	Impact Analysis		
		Description and Impact Areas (*)		on CIA (**)
An insider installs passive RFID in the location above a server room, router, array of integrated sensors or data center	Airport operations centre, the central network which handles all the decisions and processes from flight control to ground handlers. such assets include: - Server rooms - WI-FI routers - Integrated sensors used for airport smart monitoring - Airport data center - Passenger handling systems, - Automated vehicle identification and RFID-based asset tracking systems	Airport sensitive information and critical infrastructure exposed	R, L	C
A UAV equipped with a wireless antenna accesses communication links and captures data packages sent between Wi-Fi-connected devices and wireless sensors to extract information towards a malicious center of command.		Confidentiality breach, data exposure. Passengers’ and personnel’s personal information can be stolen	E, R, L	C, I
A UAV performs an acoustic attack recording valuable private information.		Confidentiality breach	R, L	C
A UAV performs a physical attack against CIs, if carrying an explosive payload		CI physical damage. Human injuries or loss of life. Air traffic flow and airport stops for safety precautions	E, H, M, R, L	I, A

(*) Impact areas: E = economic, H = human, M = material/service loss, R = reputation, L = legal. (**) Impact on information. CIA: C = confidentiality, I = integrity, A = availability.

**Table 8:** Impact analysis of scenario 3.

Threat/Hazard	Impacted Assets	Impact Analysis		
		Description and Impact Areas (*)		on CIA (**)
Attacker is equipped with an ADS-B tracing system and receives traffic data from passing aircraft	Air traffic control, secondary surveillance system (ADS-B data), aircraft safety during take-off and landing phases, separation minima, aviation safety rules	Surveillance and ATM systems’ confidentiality is compromised	L	C
Attacker injects a single UAV into the airspace, with ADS-B spoofed identity to create confusion in airport’s surveillance system and ATM		Surveillance integrity is compromised and air traffic can be disrupted or downgraded for safety reasons	E, H, M, R, L	I, A
Attacker injects a SWARM of drones equipped with ADS-B systems to create confusion in the airport’s surveillance system and ATM		Aircraft safety is jeopardized and separation minima are violated. A serious accident with aircraft may cause fatalities and serious destruction of airport’s CI	E, H, M, R, L	I, A
Attacker launches a physical attack against passing aircraft during take-off or landing			E, H, M, R, L	

(*) Impact areas: E = economic, H = human, M = material/service loss, R = reputation, L = legal. (**) Impact on information. CIA: C = confidentiality, I = integrity, A = availability.

## References

[1] European Union Artificial Intelligence and Civil Law; Liability Rules for Drones. https://www.europarl.europa.eu/thinktank/en/document.html?reference=IPOL_STU.

[2] FAA Aerospace Forecasts Unmanned Aircraft Systems. https://www.faa.gov/data_research/aviation/aerospace_forecasts/media/unmanned_aircraft_systems.pdf.

[3] United Nations Security Council Counter-Terrorism Committee Executive Directorate (CTED) Report, Greater Efforts Needed to Address the Potential Risks Posed by Terrorist Use of UAS. https://www.un.org/sc/ctc/wp-content/uploads/2019/05/CTED-UAS-Trends-Alert-Final_17_May_2019.pdf.

[4] Market Forecast, 2020 Counter Drone Market Worth USD 20 Billion. https://www.unmannedairspace.info/counter-uas-systems-and-policies/2020-counter-drone-market-worth-usd20-billion-says-market-forecast/.

[5] Nassi B., Shabtai A., Masuoka R., Elovici Y. (2019). SoK—Security and Privacy in the Age of Drones: Threats, Challenges, Solution Mechanisms and Scientific Gaps. Comput. Sci. Cryptogr. Secur..

[6] Tezza D., Andujar M. (2019). The State-of-the-Art of Human–Drone Interaction: A Survey. IEEE Access.

[7] Alsamhi S.H., Ma O., Ansari M.S., Almalki F.A. (2019). Survey on Collaborative Smart Drones and Internet of Things for Improving Smartness of Smart Cities. IEEE Access.

[8] Calantropio A. (2019). The Use of UAVs for Performing Safety-Related Tasks at Post-Disaster and Non-Critical Construction Sites. Safety.

[9] Solodov A., Williams A., Hanaei S., Goddard B. Analyzing the Threat of Unmanned Aerial Vehicles to Nuclear Facilities, SAND 2017-3408J. https://www.osti.gov/pages/servlets/purl/1356834.

[10] PARAS (2019). Guidance for Integrating Unmanned Aircraft Systems (UAS) into Airport Security. https://www.sskies.org/images/uploads/subpage/PARAS_0012.UASAirportSecurityIntegration.FinalGuidebook.pdf.

[11] U.S. Government Accountability Office (GAO 2018) Unmanned Aircraft Systems. https://www.gao.gov/key_issues/unmanned_aerial_systems/issue_summary.

[12] Altawy R., Youssef A. (2016). Security, Privacy, and Safety Aspects of Civilian Drones: A Survey. ACM Trans. Cyber-Phys. Syst..

[13] (2017). UK Dep. For Transport, Small Remotely Piloted Aircraft Systems (drones) Mid-Air Collision Study. https://assets.publishing.service.gov.uk/government/uploads/system/uploads/attachment_data/file/628092/small-remotely-piloted-aircraft-systems-drones-mid-air-collision-study.pdf.

[14] Wild G., Murray J., Baxter G. (2016). Exploring Civil Drone Accidents and Incidents to Help Prevent Potential Air Disasters. Aerospace.

[15] (2019). FAA, UAS Sightings Report, Public Records. https://www.faa.gov/uas/resources/public_records/uas_sightings_report/.

[16] NASA ASRS Database. https://asrs.arc.nasa.gov/search/database.html.

[17] Dedrone, Worldwide Drone Incidents. https://www.dedrone.com/resources/incidents/all.

[18] ASN Aviation Safety Database. https://aviation-safety.net/database/.

[19] UK Telegraph Gatwick Drone Chaos Continues into a Third Day. https://www.telegraph.co.uk/news/2018/12/20/gatwick-chaos-drones-cause-flights-cancelled-live-updates/.

[20] BBC News Heathrow Airport: Drone Sighting Halts Departures. https://www.bbc.com/news/uk-46803713.

[21] TheTelegraph Dublin Airport Forced to Suspend All Flights due to Drone Sighting over Airfield. https://www.telegraph.co.uk/news/2019/02/21/dublin-airport-drone-sighting-airfield-suspends-flights/.

[22] The Local, 143 Flights Cancelled at Frankfurt Airport due to Drone Sighting Incident. https://www.thelocal.de/20190509/disruption-after-frankfurt-airport-halts-flights-due-to-drone-sighting.

[23] Yu E. ZDNet. Singapore Changi Airport Shuts Runway over Drone Sighting. https://www.zdnet.com/article/singapore-changi-airport-shuts-runway-over-drone-sighting/.

[24] UAE Dubai Airport Airspace Closed due to Unauthorized Drone Activity. https://www.thenational.ae/uae/dubai-airport-airspace-closed-due-to-unauthorised-drone-activity-1.200601.

[25] Possible Drone Sighting Temporarily Closes Kansai International Airport. https://www.japantimes.co.jp/news/2019/10/19/national/possible-drone-sighting-temporarily-closes-kansai-international-airport/#.XhhCV_4zaUk.

[26] BBC News Drone Collides with Commercial Airplane in Canada. https://www.bbc.com/news/technology-41635518.

[27] The NewYork Times Newark Airport Traffic Halted after Drone is Spotted. https://www.nytimes.com/2019/01/22/nyregion/drones-newark-airport-ground-stop.html.

[28] WCBS-TV Drone Hits Army Helicopter Flying Over Staten Island. https://newyork.cbslocal.com/2017/09/22/drone-hits-army-helicopter/.

[29] Bloomberg South Carolina Cash Incident. https://www.bloomberg.com/news/articles/2018-02-16/what-may-be-first-drone-linked-copter-crash-being-investigated.

[30] FAA Integration of Civil Unmanned Aircraft Systems (UAS) in the National Airspace System (NAS) Roadmap. https://www.faa.gov/uas/resources/policy_library/media/Second_Edition_Integration_of_Civil_UAS_NAS_Roadmap_July%202018.pdf.

[31] Google Scholar Search Engine for Academic Publications Containing “C-UAS” Term since 2014. https://scholar.google.gr/scholar?q=c-uas&hl=el&as_sdt=1%2C5&as_vis=1&as_ylo=2014&as_yhi=2020.

[32] Stevens M., Atkins E. Geofencing in Immediate Reaches Airspace for Unmanned Aircraft System Traffic Management. Proceedings of the AIAA Information Systems-AIAA Infotech Aerospace.

[33] Hayhurst K., Maddalon J., Neogi N., Verstynen H. Case Study for Assured Containment. Proceedings of the International Conference on Unmanned Aircraft Systems (ICUAS).

[34] Stevens M., Coloe B., Atkins E. Platform-Independent Geofencing for Low Altitude UAS Operations. Proceedings of the 15th AIAA Aviation Technology, Integration, and Operations Conference.

[35] Stevens M., Atkins E. Multi-Mode Guidance for an Independent Multicopter Geofencing System. Proceedings of the 16th AIAA Aviation Technology, Integration, and Operations Conference.

[36] Zhu G., Wei P. Low-Altitude UAS Traffic Coordination with Dynamic Geofencing. Proceedings of the 16th AIAA Aviation Technology, Integration and Operations Conference.

[37] (2016). DJI, Home, Online Webpage. https://www.dji.com/phantom-4/infohttp://www.dji.com/.

[38] AISC What Is Geofencing. https://www.aisc.aero/what-is-geofencing/.

[39] DJI Enhances Geofencing Technology to Protect Airports. https://www.dji.com/ae/newsroom/news/dji-improves-geofencing-to-enhance-protection-of-european-airports-and-facilities.

[40] Skolnik I.M. (1990). Radar Handbook.

[41] Drozdowicz J., Wielgo M., Samczynski P., Kulpa K., Krzonkalla J., Mordzonek M., Bryl M., Jakielaszek Z. (2016). 35GHz FMCW drone detection system, in Radar Symposium (IRS). Proceedings of the 2016 17th International.

[42] Haag M., Bartone C., Braasch M. Flight-test evaluation of small form-factor Lidar and Radar sensors for sUAS detect-and avoid applications. Proceedings of the Digital Avionics Systems Conference (DASC), 35th IEEE/AIAA.

[43] Samaras S., Diamantidou E., Ataloglou D., Sakellariou N., Vafeiadis A., Magoulianitis V., Lalas A., Dimou A., Zarpalas D., Votis K. (2019). Deep Learning on Multi Sensor Data for Counter UAV Applications—A Systematic Review. Sensors.

[44] Wit J.M., Harmanny R., Premel-Cabic G. Micro-Doppler analysis of small UAVs. Proceedings of the 2012 9th European Radar Conference.

[45] Harmanny R., De Wit J., Cabic G. Radar micro-Doppler feature extraction using the spectrogram and the cepstrogram. Proceedings of the 2014 11th European Radar Conference.

[46] Ritchie M., Fioranelli F., Griffiths H., Torvik B. Micro-drone RCS analysis. Proceedings of the 2015 IEEE Radar Conference.

[47] Molchanov P., Harmanny R.I., de Wit J.J., Egiazarian K., Astola J. (2014). Classification of small UAVs and birds by micro-Doppler signatures. Int. J. Microw. Wirel. Technol..

[48] Fioranelli F., Ritchie M., Griffiths H., Borrion H. (2015). Classification of loaded/unloaded micro-drones using multistatic radar. Electron. Lett..

[49] Hoffmann F., Ritchie M., Fioranelli F., Charlish A., Griffiths H. Micro-Doppler based detection and tracking of UAV with multistatic radar. Proceedings of the 2016 IEEE Radar Conference (RadarConf).

[50] Zhang P., Yang L., Chen G., Li G. Classification of drones based on micro-Doppler signatures with dual-band radar sensors. Proceedings of the 2017 Progress in Electromagnetics Research Symposium-Fall (PIERS-FALL).

[51] Knott E., Schaeffer J., Tulley M. (2004). Radar Cross Section.

[52] Michel A. (2019). Counter Drone Systems.

[53] Joint Air Power Competence Centre A Comprehensive Approach to Countering Unmanned Aircraft Systems. https://www.japcc.org/portfolio/a-comprehensive-approach-to-countering-unmanned-aircraft-systems/.

[54] MyDefence, Communication White Paper, Protecting Airports against Drones. https://mydefence.dk/2019/02/mydefence-publishes-white-paper-on-airport-drone-protection/.

[55] Birnbach S., Baker R., Martinovic I. (2017). Wi-fly: Detecting Privacy Invasion Attacks by Consumer Drones.

[56] Nguyen P., Truong H., Ravindranathan M., Nguyen A., Han R., Vu T. Matthan: Drone presence detection by identifying physical signatures in the drone’rf communication. Proceedings of the 15th Annual International Conference on Mobile Systems, Applications, and Services.

[57] Nguyen P., Truong H., Ravindranathan M., Nguyen A., Han R., Vu T. (2018). Cost-effective and passive rf-based drone presence detection and characterization. GetMob. Mob. Comput. Commun..

[58] Nguyen P., Ravindranatha M., Nguyen A., Han R., Vu T. Investigating cost-effective rf-based detection of drones. Proceedings of the 2nd Workshop on Micro Aerial Vehicle Networks, Systems, and Applications for Civilian Use, ACM MobiSys 2016.

[59] (2017). Scheller, Waylon Dustin, Detecting Drones Using Machine Learning, IOWA Graduate Theses and Dissertations. https://lib.dr.iastate.edu/etd/16210.

[60] Shi Z., Huang M., Zhao C., Huang L., Du X., Zhao F. Detection of LSS UAV using hash fingerprint based SVDD. Proceedings of the 2017 IEEE International Conference on IEEE, Communications (ICC).

[61] Peacock M., Johnstone M.N. (2013). Towards Detection and Control of Civilian Unmanned Aerial Vehicles. https://ro.ecu.edu.au/cgi/viewcontent.cgi?article=1051&context=isw.

[62] Mototolea D., Stolk C. Detection and localization of small drones using commercial off-the-shelf FPGA based software defined radio systems. Proceedings of the International Conference on Communications (COMM).

[63] (2019). Blue Ribbon Task Force on UAS Mitigation at Airports, Interim Report. https://uasmitigationatairports.org/wp-content/uploads/2019/07/BRTF-Report-New-2.pdf.

[64] Chang X., Yang C., Wu J., Shi X., Shi Z. (2018). A surveillance system for drone localization and tracking using acoustic arrays. Proceedings of the IEEE 10th Sensor Array and Multichannel Signal Processing Workshop.

[65] Sedunov A., Sutin A., Sedunov N., Salloum H., Yakubovskiy A., Masters D. (2016). Passive acoustic system for tracking low-flying aircraft. IET Rada Sonar Navig..

[66] Chowdhury A. (2016). Implementation and Performance Evaluation of Acoustic Denoising Algorithms for UAV. Master’s Thesis.

[67] Mezei J., Molnár A. Drone sound detection by correlation. Proceedings of the 2016 IEEE 11th International Symposium on Applied Computational Intelligence and Informatics (SACI).

[68] Bernardini A., Mangiatordi F., Pallotti E., Capodiferro L. (2017). Drone detection by acoustic signature identification. Electron. Imaging.

[69] Mirelli V., Tenney S., Bengio Y., Chapados N., Delalleau O. (2009). Statistical machine learning algorithms for target classification from acoustic signature. Proc. MSS Battlespace Acoust. Magn. Sens..

[70] Kim J., Park C., Ahn J., Ko Y., Park J., Gallagher J.C. Real-time UAV sound detection and analysis system. Proceedings of the 2017 IEEE Sensors Applications Symposium (SAS).

[71] Salamon J., Jacoby C., Bello J.P. A dataset and taxonomy for urban sound research. Proceedings of the 22nd ACM International Conference on Multimedia.

[72] Jeon S., Shin J., Lee Y., Kim W., Kwon Y., Yang H. Empirical study of drone sound detection in real-life environment with deep neural networks. Proceedings of the 2017 25th European Signal Processing Conference (EUSIPCO).

[73] Park S., Shin S., Kim Y., Matson E.T., Lee K., Kolodzy P.J., Slater J.C., Scherreik M., Sam M., Gallagher J.C. Combination of radar and audio sensors for identification of rotor-type unmanned aerial vehicles (uavs). Proceedings of the 2015 IEEE SENSORS.

[74] Hu S., Goldman G., Borel-Donohue C. (2017). Detection of unmanned aerial vehicles using a visible camera system. Appl. Opt..

[75] Rozantsev A., Lepetit V., Fua P. Flying objects detection from a single moving camera. Proceedings of the IEEE Conference on Computer Vision and Pattern Recognition.

[76] Santana L., Brandao A., Sarcinelli-Filho M., Carelli R. A trajectory tracking and 3d positioning controller for the ar. Drone quadrotor. Proceedings of the Unmanned Aircraft Systems (ICUAS), International Conference on IEEE.

[77] Unlu E., Zenou E., Rivière N. (2018). Using shape descriptors for UAV detection. Electron. Imaging.

[78] Saqib M., Khan S., Sharma N., Blumenstein M. A study on detecting drones using deep convolutional neural networks. Proceedings of the 14th IEEE International Conference on Advanced Video and Signal Based Surveillance (AVSS).

[79] Aker C., Kalkan S. (2017). Using Deep Networks for Drone Detection. arXiv.

[80] Unlu E., Zenou E., Rivière N. Generic Fourier descriptors for autonomous UAV detection. Proceedings of the 7th International Conference on Pattern Recognition Applications and Methods (ICPRAM2018).

[81] Rozantsev A., Sinha S., Dey D., Fua P. Flight dynamics-based recovery of a UAV trajectory using ground cameras. Proceedings of the Conference Computer Vision and Pattern Recognition.

[82] Opromolla R., Fasano G., Accardo D. (2018). A Vision-Based Approach to UAV Detection and Tracking in Cooperative Applications. Sensors.

[83] Gökçe F., Üçoluk G., ¸Sahin E., Kalkan S. (2015). Vision-based detection and distance estimation of micro unmanned aerial vehicles. Sensors.

[84] Freitas S., Silva H., Almeida J., Silva E. (2018). Hyperspectral imaging for real-time unmanned aerial vehicle maritime target detection. J. Intell. Robot. Syst..

[85] Pham T., Takalkar M., Xu M., Hoang D., Truong H., Dutkiewicz E., Perry S. Airborne Object Detection Using Hyperspectral Imaging: Deep Learning Review. Proceedings of the International Conference on Computational Science and Its Applications.

[86] Müller T. Robust drone detection for day/night counter-UAV with static VIS and SWIR cameras. Proceedings of the Ground/Air Multisensor Interoperability, Integration, and Networking for Persistent ISR VIII, International Society for Optics and Photonics.

[87] Birch G.C., Woo B.L. (2017). Counter unmanned aerial systems testing: Evaluation of VIS SWIR MWIR and LWIR passive imagers. Sandia Rep..

[88] Thomas A., Cotinat A., Gilber M. UAV localization using panoramic thermal cameras. Proceedings of the 12th International Conference on Computer Vision Systems (ICVS).

[89] Church P., Grebe C., Matheson J., Owens B. Aerial and surface security applications using Lidar. Proceedings of the Laser Radar Technology and Applications XXIII, International Society for Optics and Photonics.

[90] FAA Unmanned Aircraft System Traffic Management (UTM). https://www.faa.gov/uas/research_development/traffic_management/.

[91] Luo A. Drones hijacking—multi-dimensional attack vectors and countermeasures. Presented at the DEF CON 24 Conference.

[92] Robinson M. Knocking My Neighbors Kids Cruddy Drone Offline. DEF CON 23. https://academic.csuohio.edu/yuc/mobile/GPS-Knocking-My-Neighbors-Kid-Drone-compressed.pdf.

[93] Rodday N. Hacking a professional drone. Proceedings of the Black Hat ASIA 2016.

[94] Highnam K., Angstadt K., Leach K., Weimer W., Paulos A., Hurley P. An uncrewed aerial vehicle attack scenario and trustworthy repair architecture. Proceedings of the 46th Annual IEEE/IFIP International Conference on Dependable Systems and Networks Workshop (DSN-W).

[95] Davidson D., Wu H., Jellinek R., Singh V., Ristenpart T. Controlling UAVs with sensor input spoofing attacks. Proceedings of the 10th USENIX Workshop on Offensive Technologies (WOOT).

[96] Kerns A., Shepard D., Bhatti J., Humphreys T. (2014). Unmanned aircraft capture and control via GPS spoofing. J. Field Robot..

[97] Feng Z., Guan N., Lv M., Liu W., Deng Q., Liu X., Yi W. Efficient drone hijacking detection using onboard motion sensors. Proceedings of the Design, Automation & Test in Europe Conference & Exhibition (DATE).

[98] Tippenhauer N., Pöpper C., Rasmussen K., Capkun S. On the requirements for successful GPS spoofing attacks. Proceedings of the 18th ACM Conference on Computer and Communications Security.

[99] Vervisch-Picois A., Samama N., Taillandier-Loize T. Influence of GNSS spoofing on drone in automatic flight model. Proceedings of the ITSNT 2017: 4th International Symposium of Navigation and Timing. Ecole Nationale de L’aviation Civile (ENAC).

[100] He D., Qiao Y., Chen S., Du X., Chen W., Zhu S., Guizani M. (2018). A friendly and low-cost technique for capturing non-cooperative civilian unmanned aerial vehicles. IEEE Netw..

[101] Humphreys T.E. Statement on the Vulnerability of Civil Unmanned Aerial Vehicles and Other Systems to Civil GPS Spoofing. http://homeland.house.gov/sites/homeland.house.gov/files/Testimony-Humphreys.pdf.

[102] Mitch R., Dougherty R., Psiaki M., Powell S.P., O’Hanlon B.W. (2011). Signal Characteristics of Civil GPS Jammers. https://repositories.lib.utexas.edu/bitstream/handle/2152/63304/Signal%20Characteristics%20Civil%20GPS%20Jammers_Mitch.pdf?sequence=2.

[103] U.S. Dept. of Homeland Security, Science and Technology Directorate, National Urban Security Technology Laboratory (NUSTL) (2019). Counter-Unmanned Aircraft Systems Technology Guide. https://www.dhs.gov/sites/default/files/publications/c-uas-tech-guide_final_28feb2020.pdf.

[104] Michel A. (2018). Counter-Drone Systems.

[105] Unmanned Airspace Information Portal, Counter-UAS Industry Directory. https://www.unmannedairspace.info/counter-uas-industry-directory/.

[106] Lykou G., Anagnostopoulou A., Gritzalis D. (2019). Smart Airports Cybersecurity: Threat Mitigation and Cyber Resilience, SENSORS. https://www.mdpi.com/1424-8220/19/1/19.

[107] Lykou G., Anagnostopoulou A., Gritzalis D. Implementing cyber-security measures in airports to improve cyber-resilience. Proceedings of the Workshop on Industrial Internet of Things Security (WIIoTS-2018).

[108] Lykou G., Iakovakis G., Gritzalis D., Gritzalis D., Theoharidou M., Stergiopoulos G. (2019). Aviation Cybersecurity and Cyber-Resilience: Assessing Risk in Air Traffic Management. Critical Infrastructure Security and Resilience.

[109] Business Standard, Houthi says it targeted Saudi Arabia’s Abha Airport with Drone Attack. https://www.business-standard.com/article/news-ani/houthi-says-it-targeted-saudi-arabia-s-abha-airport-with-drone-attack-119072900175_1.html.

[110] The Guardian, Middle East Drones Signal End to era of Fast jet air Supremacy. https://www.theguardian.com/world/2019/sep/16/middle-east-drones-signal-end-to-era-of-fast-jet-air-supremacy.

[111] ICAO, Annex 10, Aeronautical Telecommunications. https://store.icao.int/collections/annex-10-aeronautical-telecommunications.

[112] SESAR JU, Addressing Airport Cyber-Security. https://www.sesarju.eu/sites/default/files/documents/news/Addressing_airport_cyber-security_Full_0.pdf.

[113] Lupu T.G., Rudas I., Demiralp M., Mastorakis N. (2009). Main Types of Attacks in Wireless Sensor Networks. http://www.wseas.us/e-library/conferences/2009/budapest/MIV-SSIP/MIV-SSIP31.pdf.

[114] Wilkinson G. Digital Terrestrial Tracking: The Future of Surveillance. https://pdfs.semanticscholar.org/07a5/08ddd6cc3eadd1f0743e7acf8a38db467703.pdf.

[115] Gittleson K. Data-Stealing Snoopy Drone Unveiled at Black Hat. https://www.bbc.com/news/technology-26762198.

[116] Karanam C., Mostofi Y. 3D Through-Wall Imaging with Unmanned Aerial Vehicles Using WiFi. https://www.ece.ucsb.edu/~ymostofi/papers/IPSN17_KaranamMostofi.pdf.

[117] Guri M., Zadov B., Elovici Y. (2017). Led-it-Go: LEAKING (a lot of) Data from Air-Gapped Computers via the (Small) Hard Drive Led. International Conference on Detection of Intrusions and Malware, and Vulnerability Assessment.

[118] Kleiner A., Prediger J., Nebel B. RFID Technology-based Exploration and SLAM for Search and Rescue. Proceedings of the RSJ International Conference on Intelligent Robots and Systems.

[119] Magnabosco M., Breckon T.P. (2013). Cross-Spectral Visual Simultaneous Localization And Mapping (SLAM) with Sensor Handover. Rob. Auton. Syst..

[120] Karlsson N., Di Bernardo E., Ostrowski J., Goncalves L., Pirjanian P., Munich M.E. The vSLAM Algorithm for Robust Localization and Mapping. Proceedings of the 2005 IEEE International Conference on Robotics and Automation.

[121] Moore J. (2019). Drone de-Confliction Advances ADS-B for UAS Benefits All Airspace Users, AOPA. https://www.aopa.org/news-and-media/all-news/2019/may/22/drone-deconfliction-advances.

[122] Costin A., Francillon A. Ghost in the Air(Traffic): On Insecurity of ADS-B Protocol and Practical Attacks on ADS-B Devices. https://www.researchgate.net/publication/267557712_Ghost_in_the_AirTraffic_On_insecurity_of_ADS-B_protocol_and_practical_attacks_on_ADS-B_devices.

[123] Mohsen M., Kaabouch N. (2017). Analysis of vulnerabilities, attacks, countermeasures and overall risk of the Automatic Dependent Surveillance-Broadcast (ADS-B) system. Int. J. Crit. Infrastruct. Prot..

[124] Santamarta R. (2018). Last Call for SATCOM Security.

[125] FAA (2018). Letter from FAA Office of Airports on Guidance on Use of Counter UAS Systems at Airports. https://www.faa.gov/airports/airport_safety/media/attachment-1-counter-uas-airport-sponsor-letter-july-2018.pdf.

